# A modified vaccinia Ankara vaccine expressing spike and nucleocapsid protects rhesus macaques against SARS-CoV-2 delta infection

**DOI:** 10.1126/sciimmunol.abo0226

**Published:** 2022-03-31

**Authors:** Nanda Kishore Routhu, Sailaja Gangadhara, Lilin Lai, Meredith Elizabeth Davis Gardner, Katharine Floyd, Ayalnesh Shiferaw, Yannic C Bartsch, Stephanie Fischinger, Georges Khoury, Sheikh Abdul Rahman, Samuel David Stampfer, Alexandra Schaefer, Sherrie M. Jean, Chelsea Wallace, Rachelle L. Stammen, Jennifer Wood, Joyce Cohen, Tamas Nagy, Matthew S. Parsons, Lisa Gralinski, Pamela A. Kozlowski, Galit Alter, Mehul S. Suthar, Rama Rao Amara

**Affiliations:** ^1^ Division of Microbiology and Immunology, Emory Vaccine Center, Yerkes National Primate Research Center, Emory University, Atlanta, Georgia 30329, USA.; ^2^ Department of Microbiology and Immunology, Emory School of Medicine, Emory University, Atlanta, Georgia 30322, USA.; ^3^ Ragon Institute of MGH, MIT and Harvard, Cambridge, Massachusetts 02139, USA.; ^4^ Division of Animal Resources, Yerkes National Primate Research Center, Emory University, Atlanta, Georgia 30329, USA.; ^5^ College of Veterinary Medicine, University of Georgia, Athens, Georgia 30602, USA.; ^6^ Department of Pathology and Laboratory Medicine, Emory University School of Medicine, Atlanta, GA, USA.; ^7^ Department of Epidemiology, University of North Carolina, Chapel Hill, North Carolina 27516, USA; ^8^ Department of Microbiology, Immunology, and Parasitology, Louisiana State University Health Sciences Center, New Orleans, Louisiana 70112, USA.; ^9^ Department of Pediatrics, Division of Infectious Diseases, Emory University School of Medicine, Atlanta, GA 30322, USA.

## Abstract

SARS-CoV-2 vaccines should induce broadly cross-reactive humoral and T cell responses to protect against emerging variants of concern (VOCs). Here, we inactivated the furin-cleavage site (FCS) of spike expressed by a modified vaccinia Ankara (MVA) virus vaccine (MVA/SdFCS) and found that FCS inactivation markedly increased spike binding to human ACE2. Following vaccination of mice, the MVA/SdFCS vaccine induced 8-fold higher neutralizing antibodies compared to MVA/S, which expressed spike without FCS inactivation, and protected against the beta variant. We next added nucleocapsid to the MVA/SdFCS vaccine (MVA/SdFCS-N) and tested its immunogenicity and efficacy via intramuscular (IM), buccal (BU) or sublingual (SL) routes in rhesus macaques. IM vaccination induced spike-specific IgG in serum and mucosae (nose, throat, lung, rectum) which neutralized the homologous (WA-1/2020) and heterologous VOCs, including delta, with minimal loss (<2-fold) of activity. IM vaccination also induced both S and N specific CD4 and CD8 T cell responses in the blood. In contrast, the SL and BU vaccinations induced less spike-specific IgG in secretions and lower levels of polyfunctional IgG in serum compared to IM vaccination. Following challenge with SARS-CoV-2 delta variant, the IM route induced robust protection, BU moderate protection and the SL no protection. Vaccine-induced neutralizing and non-neutralizing antibody effector functions positively correlated with protection, but only the effector functions correlated with early protection. Thus, IM vaccination with MVA/SdFCS-N vaccine elicited cross-reactive antibody and T cell responses, protecting against heterologous SARS-CoV-2 VOC more effectively than other routes of vaccination.

## Teaser

An MVA-based spike and nucleocapsid vaccine induced protective antibody and T cell responses against SARS-CoV-2 delta infection.

## INTRODUCTION

Coronavirus disease 2019 (COVID-19), caused by severe acute respiratory syndrome coronavirus type 2 (SARS-CoV-2), emerged in late 2019 with subsequent rapid spread throughout the world (*1*). It has caused devastating morbidity, mortality, and economic damage worldwide despite stringent intervention strategies and rapid vaccine development (*2*). SARS-CoV-2 has infected more than a quarter billion people resulting in over 5 million deaths worldwide (*3*). Operation Warp Speed yielded rapid development of mRNA technology-based (Moderna and Pfizer/BioNTech) and viral vector-based (Oxford-AstraZeneca and Janssen and Janssen) vaccines, which induce strong neutralizing antibodies and dramatically reduce hospitalizations and mortality (*4*). Both mRNA-based vaccines, mRNA-1273 and BNT162b2 have shown ~95% efficacy in clinical trials when the circulating SARS-CoV-2 strains are the wild-type WA-1/2020 strain and near-wild-type D614G variant (*5*, *6*).

Most of the current first-generation vaccines utilize the prefusion-stabilized forms of spike protein derived from WA-1/2020 strain to generate highly potent autologous SARS-CoV-2 neutralizing antibodies in preclinical animal models and vaccinated individuals (*7*–*10*). Subsequently, mutations have accumulated in the SARS-CoV-2 genome resulting in the emergence of novel variants of concern (VOCs) (*2*). Some mutations confer fitness advantages through improved viral replication, higher transmissibility, and immune evasion. Of particular interest are mutations in the spike protein and in its receptor-binding domain (RBD), which determine the ability of the VOC to evade the vaccine-induced immune response (*11*). VOCs such as B.1.1.7 (Alpha, isolated in UK), B.1.351 (Beta, isolated in South Africa), P.1 (Gamma, isolated in Brazil) and B.1.1.529 (Omicron, isolated in South Africa) have variable decreases in neutralizability, transmissibility, and pathogenicity. Of particular interest is the B.1.617.2 variant (Delta, isolated in India), which is responsible for widespread infections in both vaccinated and unvaccinated people at the time this study was conducted (*2*). Delta spike protein-specific substitutions include T19R, Δ157-158, L452R, T478K, D614G, P681R, and D950N (*2*), resulting in efficient membrane fusion and reduced sensitivity to neutralizing antibodies derived from individuals who had recently received two doses of mRNA-based COVID-19 vaccine (*12*–*14*). A third dose (booster) of mRNA vaccine increases anti-spike antibody levels and provides additional clinical protection against COVID-19 disease and hospitalization (*15*, *16*).

While the current booster approach reduces COVID-19 severity in the short term, the incomplete protection provided by current vaccines, particularly against VOCs, warrants the development of vaccines with more durable immunity. For instance, the neutralizing antibodies induced by spike protein from the Washington strain show diminished cross-reactivity against some of the VOC such as beta, delta, and omicron (*17*, *18*). In particular, the cross-reactivity to omicron is reduced greater than 20-fold after two mRNA vaccinations (*19*). In contrast, a significant fraction of T cell epitopes are conserved across multiple human betacoronaviruses (*20*, *21*). One strategy is to devise a vaccine that induces broadly-reactive B and T cell responses that retain activity even as progressive mutations in spike protein result in escape from common neutralizing antibodies (*22*, *23*). T cells react with epitopes that may be distinct from those commonly bound by antibodies and may retain activity against such future variants. While spike continues to accumulate mutations, the nucleocapsid (N) protein remains significantly more conserved among betacoronaviruses (*24*). It is a major structural protein and induces potent T cell responses during natural infection (*25*–*27*). For these reasons, it is an attractive target for induction of broadly-active T cells. Additionally, T cells can also provide localized protection in the respiratory mucosa so enhancing mucosal immunity could also prevent new infections by destroying incoming virions before they can replicate, reduce disease severity via local antiviral effects in the lower respiratory tract, and stop transmission by damaging virions prior to exhalation. All current licensed COVID-19 vaccines are delivered intramuscularly, but vaccination via a mucosal route is an attractive option to induce formation of mucosal antibodies and T cells. Thus, the development of COVID-19 vaccines that co-express spike (S) and N, and their delivery via mucosal route is important.

We previously evaluated a modified vaccinia Ankara (MVA)-based SARS-CoV-2 vaccine (MVA/S) expressing the full-length spike with two proline mutations (K986P/V987P, S-2P) to stabilize in its prefusion conformation. We showed that the MVA/S vaccine was highly immunogenic in mice and was able to protect against WA-1/2020 infection in rhesus macaques (*28*). In this study we investigated the effectiveness of an improved MVA-based SARS-CoV-2 vaccine expressing S with furin-cleavage site inactivation (SdFCS) and N proteins, evaluating its immunogenicity and efficacy when administered via either intramuscular (IM) or oral needle-free route against heterologous SARS-CoV-2 challenge in rhesus macaques. For oral immunizations we used either buccal or sublingual site. Our results showed differences in the antibody responses induced by different routes of vaccination and revealed correlates for vaccine protection against the heterologous delta VOC.

## RESULTS

### Furin-cleavage site inactivation improves cell surface expression and ACE2 binding of spike-2P expressed by MVA vaccine

**Fig. 1.**
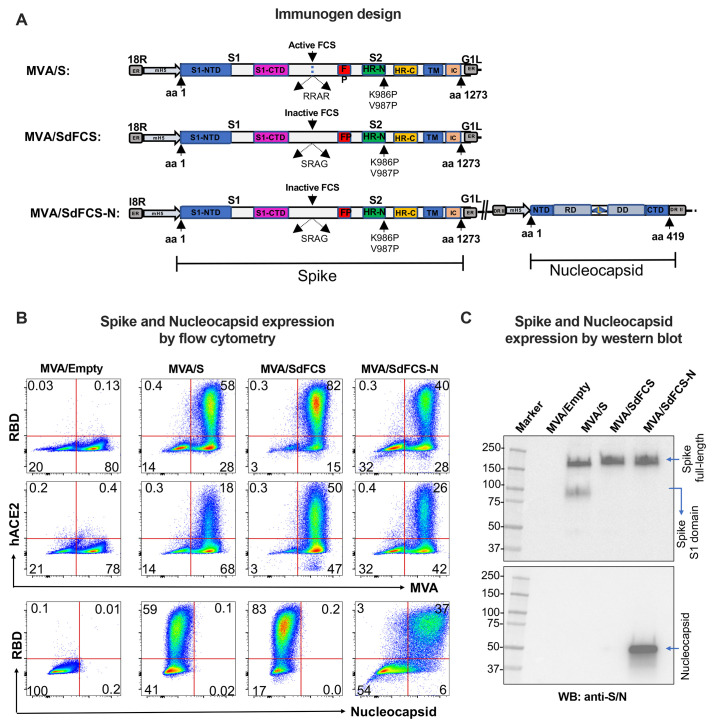
Schematics designs and characterization of recombinant Modified Vaccinia Ankara (MVA)-based SARS-CoV-2 vaccines expression. (**A**) Domains that represented in MVA/S, MVA/SdFCS, and MVA/SdFCS-N constructs are colored. Recombinant spike (S) and Nucleocapsid (N) inserts were cloned in the essential region (ER) in between I8R and G1L, and deletion region II (DR II) respectively, under modified H5 (mH5) promoter. NTD – N terminal domain; CTD – C terminal domain; FP – Fusion peptide; HR-N – Heptad repeat N; HR-C – Heptad repeat C; TM – Transmembrane anchor; IC – intracellular tail; Active FCS (FCS – Furin cleavage site – RRAR); Inactive FCS (FCS mutation – SRAG); RD – RNA binding domain; L – Linker; and DD – Dimerization domain. Arrows represents amino acid numbers and protease cleavage sites. (**B**) MVA/S (carrying S-2P), MVA/SdFCS (carrying S-2P and furin-cleavage site-inactivated), and MVA/SdFCS-N (SdFCS with N) vaccines showing the spike expression and ACE2 binding in infected-DF-1 cells by flow cytometry. (**C**) MVA/S, MVA/SdFCS and MVA/SdFCS-N vaccines showing the spike and N expression in infected-DF-1 cells in Western blotting analysis.

Previous studies show full-length spike protein (amino acids 1-1273) can be stabilized in its prefusion conformation via introduction of two proline mutations K986P/V987P (*8*, *10*). Accordingly, we introduced these two proline mutations into spike protein expressed by MVA (MVA/S-2P) and showed high levels of spike surface expression and human ACE2 binding (*28*). To further stabilize surface expression of S-2P, we inactivated its FCS at amino acids 682-685 by mutating RRAR to SRAG and generated the corresponding MVA vaccine, MVA/SdFCS (**Fig. 1A** **,** **1B**). We evaluated spike surface expression and binding to ACE2 by flow cytometry and found that inactivation of FCS further enhanced both activities (**Fig. 1B**). As expected, the SdFCS protein was not cleaved based on Western blot analysis (**Fig. 1C**). These results are consistent with a previous study showing better binding of membrane anchored spike to anti-RBD antibody and ACE2 following the inactivation of FCS in the backbone of spike with proline mutations (*29*). However, the previous study did not address the impact of FCS inactivation in the background of 2P mutation on the immunogenicity and protection. We next generated MVA/SdFCS-N vaccine by adding the N gene under the control of mH5 promoter in deletion II region of MVA/SdFCS vaccine (**Fig. 1A**). The MVA/SdFCS-N vaccine also expressed high levels of spike that bound strongly to human ACE2, and the N protein (**Fig. 1, B** and **C**). We observed a marginally lower expression of spike in the MVA/SdFCS-N vaccine compared to MVA/SdFCS vaccine (**Fig. 1B**).

### MVA/SdFCS elicits greater humoral immune responses than MVA/S and protects against homologous and heterologous SARS-CoV-2 challenges in mice

**Fig. 2.**
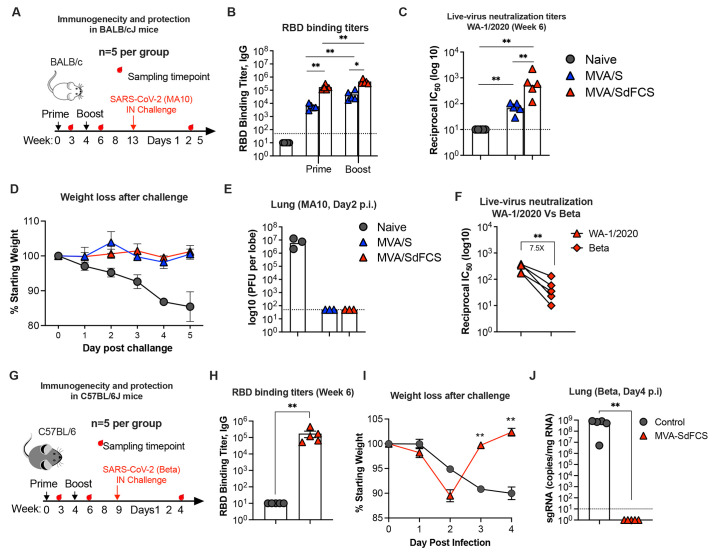
MVA/SdFCS vaccine protects from SARS-CoV-2 WA-1/2020 and beta (B.1.351) infection in mice. (**A-E**) Immunogenicity and protection in BALB/cJ mice against SARS-CoV-2 (WA-1/2020) challenge. (**A**) Schematic of the BALB/cJ mouse study. Mice (5 mice per group) were immunized via intramuscular route (IM) at weeks 0 and 4 with MVA/S (blue) or MVA/SdFCS (red). Control mice received no treatment (grey). Serum collected from 3-weeks after the first immunization (post-prime) and 2-weeks after second immunization (post-boost) was used to assess RBD (WA-1/2020)-specific binding (**B**) and WA-1/2020 strain specific neutralizing (**C)** antibody response. Seven weeks post-boost, mice were intranasally (IN) challenged with 10^5 PFU of mouse-adapted SARS-CoV-2 (MA10). Percent weight loss up to day 5 (n=5 until day 2, the n=2 until day 5) (**D**) and lung viral titers (day 2 post-infection) (**E**) of vaccinated animals was compared to unvaccinated animals. (**F**) Cross-neutralizing antibody activity against beta (B.1.351) in MVA/SdFCS animals at 2 weeks after the boost. (**G-J**) Immunogenicity and protection in C57BL/6J mice against SARS-CoV-2 beta (B.1.351) challenge. (**G**) Schematic of the C57BL/6J mouse study. C57BL/6J mice (5 mice per group) were immunized via intramuscular route (IM) at weeks 0 and 4 with MVA/SdFCS (red). Control mice were administered phosphate-buffered saline (PBS) (grey). Serum collected at week 6 (2-weeks post-boost) was used to assess RBD (WA-1/2020)-specific binding antibody response (**H**). At week 9, mice were intranasally (IN) challenged with 10^5 PFU of SARS-CoV-2 beta (B.1.351). Weight loss (**I**) and lung SARS-CoV-2 beta (B.1.351) viral sub-genomic RNA (sgRNA) loads (**J**) post-infection. Each sample was analyzed in duplicates. Data are mean ± SEM. A two-sided Mann–Whitney U-test was used to compare between groups, *p < 0.05 and **p < 0.01.

To test the immunogenicity and efficacy of the MVA/SdFCS vaccine and to compare it with the MVA/S vaccine, we immunized BALB/cJ mice via the IM route with MVA/SdFCS or MVA/S vaccines using a week 0 prime and week 4 boost regimen (**Fig. 2A**). RBD binding IgG responses were analyzed at week 3 (3-weeks post-prime) and 6 (2-weeks post-boost). MVA/SdFCS immunization elicited significantly higher antibody titers following both the prime (29-fold) and boost (12-fold) timepoints compared to MVA/S (**Fig. 2B**). The RBD binding antibody titer induced after a single immunization with MVA/SdFCS was comparable to the titer induced after two immunizations with MVA/S (**Fig. 2B**). Consistent with high RBD-binding activity, the MVA/SdFCS vaccinated animals also showed 8-fold higher live-virus neutralization activity against the homologous SARS-CoV-2 (WA-1/2020) (**Fig. 2C**). To compare vaccine efficacy, we challenged mice intranasally (IN) with 10^5^ plaque-forming units (pfu) of mouse adapted SARS-CoV-2 virus (MA10) (*30*) at week 13 (day 0) and monitored body weight until day 5 (n=5 until day 2, n=2 until day 5) and viral titers in lung tissue at day 2 (n=3)(**Fig. 2, D** and **E**) after challenge. Unvaccinated animals showed very high viral titers (~10^7 pfu per lung lobe) and lost approximately 15% of their body weight by day 5. In contrast, MVA/SdFCS and MVA/S-vaccinated animals showed complete protection with no weight loss and no detectable live virus in the lungs. These data demonstrated that MVA-based SARS-CoV-2 vaccines provide protection against homologous SARS-CoV-2 virus challenge in mice.

We next determined neutralization titer against beta variant, since this VOC is harder to neutralize (*31*). Consistent with other studies, we observed a 7.5-fold reduction in live-virus neutralization titer against SARS-CoV-2 beta compared to the wild-type WA-1/2020 strain (**Fig. 2F**). This prompted us to explore the protection of our WA-1/2020-based MVA/SdFCS vaccine against heterologous SARS-CoV-2 beta viral challenge. We immunized C57BL/6J mice with MVA/SdFCS vaccine on weeks 0 and 4. We used C57BL/6J mice since we had just set up this model for beta virus challenge and MVA vaccinated mice were readily available. At week 11, the vaccinated mice and unvaccinated controls were challenged intranasally with 10^5^ pfu of SARS-CoV-2 beta virus (**Fig. 2G**). As expected, MVA/SdFCS vaccination elicited strong RBD-specific IgG antibody titers at week 6 during vaccination (geometric mean titer of 125,650)(**Fig. 2H**). Following challenge, the MVA/SdFCS-vaccinated animals showed transient weight loss at day 2 and recovered quickly at day 3 (**Fig. 2I**) and did not show any detectable sub-genomic RNA (sgRNA) in lungs at day 4 (**Fig. 2J**). In contrast, unvaccinated animals experienced a 10% weight loss by day 4 along with high lung sgRNA levels. Altogether, the MVA/SdFCS provided heterologous protection against difficult to neutralize SARS-CoV-2 beta VOC in mice.

### Intramuscular MVA/SdFCS-N vaccine induces cross-reactive binding antibody responses in systemic and mucosal compartments of macaques

**Fig. 3.**
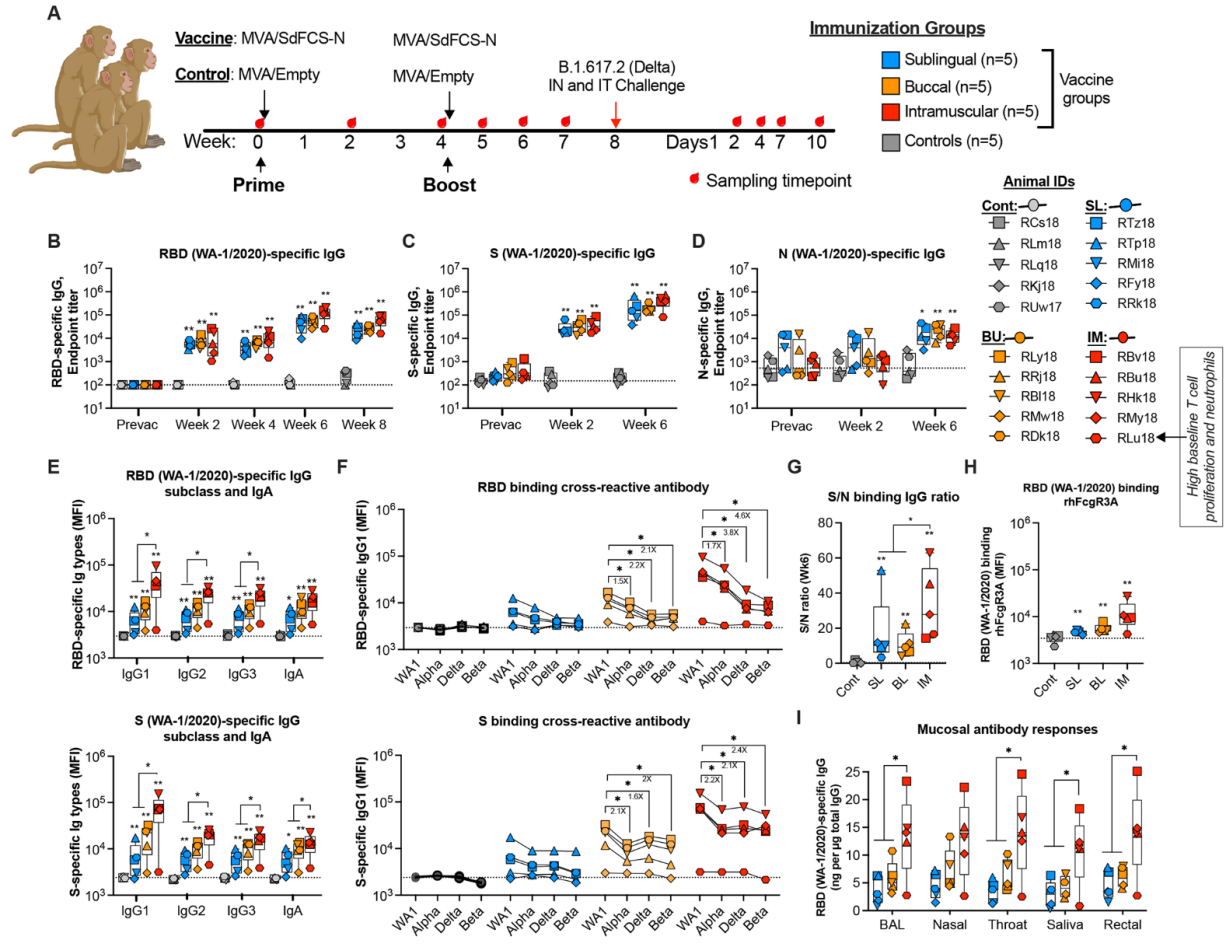
Systemic and mucosal antibody responses elicited following MVA/SdFCS-N immunization in rhesus macaques. (**A**) Schematic of immunizations and SARS-CoV-2 challenges in rhesus macaques. Twenty rhesus macaques were divided into four groups (n = 5 per group).10^8 PFU of MVA/SdFCS-N vaccine was administered via sublingual (SL, aqua blue), buccal (BU, orange) and intramuscular (IM, red) routes and 10^8 PFU of MVA/Empty was delivered via the intramuscular route (Controls, black) at weeks 0 (prime) and 4 (boost). The animals were challenged with SARS-CoV-2 delta (B.1.617.2) virus via intranasal and intratracheal (IT) routes at week 8. The sample collection and necropsy timepoints are indicated by red droplets. Sera collected at pre-vaccination (week 0) and post-vaccination (week 2, 4, 6 and 8) timepoints was used to assess for SARS-CoV-2 WA-1/2020 RBD (**B**), S (**C**), and N (**D**)-specific antibodies longitudinally. Post-boost (week 6) serum was used to assess WA-1/2020 (RBD (upper), and S (lower))-specific immunoglobulin (Ig) isotypes (**E**), and RBD (upper) and S (lower)-specific alpha, delta, and beta- cross-binding IgG1 antibody titers (**F**), respectively. (**G**) Ratio of S binding IgG to N binding IgG. (**H**) RBD (WA-1/2020) binding FcγR3A. (**I**) Post-vaccination (week 6), RBD (WA-1/2020)-specific IgG antibody was assessed in bronchoalveolar lavage (BAL) fluid, and nasal, throat, salivary, and rectal secretions. Data represent one independent experiment. Each sample was analyzed in duplicate. Individual data points reflected by unique shapes in Fig. 3B to 3I represent individual NHP. Whisker plots show the maximum and minimum values. Dotted lines indicate binding assay limits of detection. Data are mean ± SEM. A two-sided Mann–Whitney U-test was used to compare between groups, *p < 0.05 and **p < 0.01.

To increase the breadth and induce cross-reactive T cell response, we next used dual MVA recombinant vaccine expressing SdFCS and N (MVA/SdFCS-N)(**Fig. 1A** **)**. To assess the immunogenicity and efficacy of the MVA/SdFCS-N vaccine, and to test if oral needle-free immunizations induce better mucosal immunity in the upper and lower respiratory tract, we immunized rhesus macaques (RMs) (n=5/group) at weeks 0 and 4 via sublingual (SL), buccal (BU) or, intramuscular (IM) routes. Control RMs received MVA vaccine without any recombinant insert (MVA/Empty) via IM route (**Fig. 3A**). We challenged all animals with SARS-CoV-2 delta variant at week 8 to determine vaccine efficacy.

We assessed SARS-CoV-2 (WA-1/2020) RBD-, S- and N-specific humoral immune responses in serum longitudinally following vaccination (**Fig. 3, B to D**). The priming vaccination induced comparable RBD- and S-specific IgG (geometric mean titer of about 6x10^3^ and 3x10^4^, respectively for RBD and spike) in all vaccinated RMs at week 2. The titers increased by about 10-fold (geometric mean titer of 5.8x10^4^ and 2.1x10^5^ for RBD and S) at week 6 (2 weeks post boost) and were durable through week 8 in all vaccinated animals irrespective of vaccination route **(** **Fig. 3, B** and **C** **).** At their peak response, S-specific IgG titers were 3 to 4-fold higher than corresponding RBD titers. Vaccinations also induced N-specific binding antibodies mostly following the boost (geometric mean of 1-2x10^4^ at week 6) that were 10-60 times lower compared to spike binding titers (**Fig. 3D**). The NC specific antibody responses in the SL group at week 6 was not significantly different compared to pre- (week 0) or post prime- (week 2) vaccination. However, in BU and IM groups the NC specific response at week 6 was significantly higher compared to pre- (week 0) or post prime- (week 2) vaccination. Most of the IgG responses were IgG1 with some IgG2 and IgG3, indicative of a Th1-dominant response (**Fig. 3E**). Vaccinations also induced low level of RBD and spike specific IgA in serum (**Fig. 3E**). The IgG1, IgG2, IgG3 and IgA antibody also showed cross-reactive binding to RBD **(figs. S1, A to C)** and S **(figs. S1, D to F**) proteins from VOC viruses alpha, delta and beta VOC (**Fig. 3F** **).** The S binding activity was comparable between alpha, delta and beta VOCs whereas RBD binding declined with least binding to beta VOC. The ratio of S to N binding IgG ratio was significantly higher in IM vaccinated animals than orally vaccinated animals (**Fig. 3G**). The vaccine-induced RBD-specific sera also showed binding to rhesus Fc gamma receptor 3A (FcγR3A)(**Fig. 3H**). Vaccinations also induced RBD (WA-1 and delta)-specific IgG in the lower (lung), the upper airway (nose, throat) mucosal compartments, the saliva, and the rectum (**Fig. 3I** **)** (**fig. S1G**). In general, IM vaccinations induced significantly higher IgG1, IgG2, IgG3 and IgA antibodies in serum, and IgG antibodies in mucosal compartments compared to the two oral vaccination groups (**Fig. 3, E** and **I** **)**. However, both SL and BU vaccinations induced similar binding antibody to spike and N. These results demonstrated that IM vaccinations induced significantly higher binding antibody in serum and mucosal secretions compared to oral vaccinations with cross-reactivity to S proteins from other VOCs.

### Intramuscular vaccination induces poly-functional antibodies in serum of macaques

**Fig. 4.**
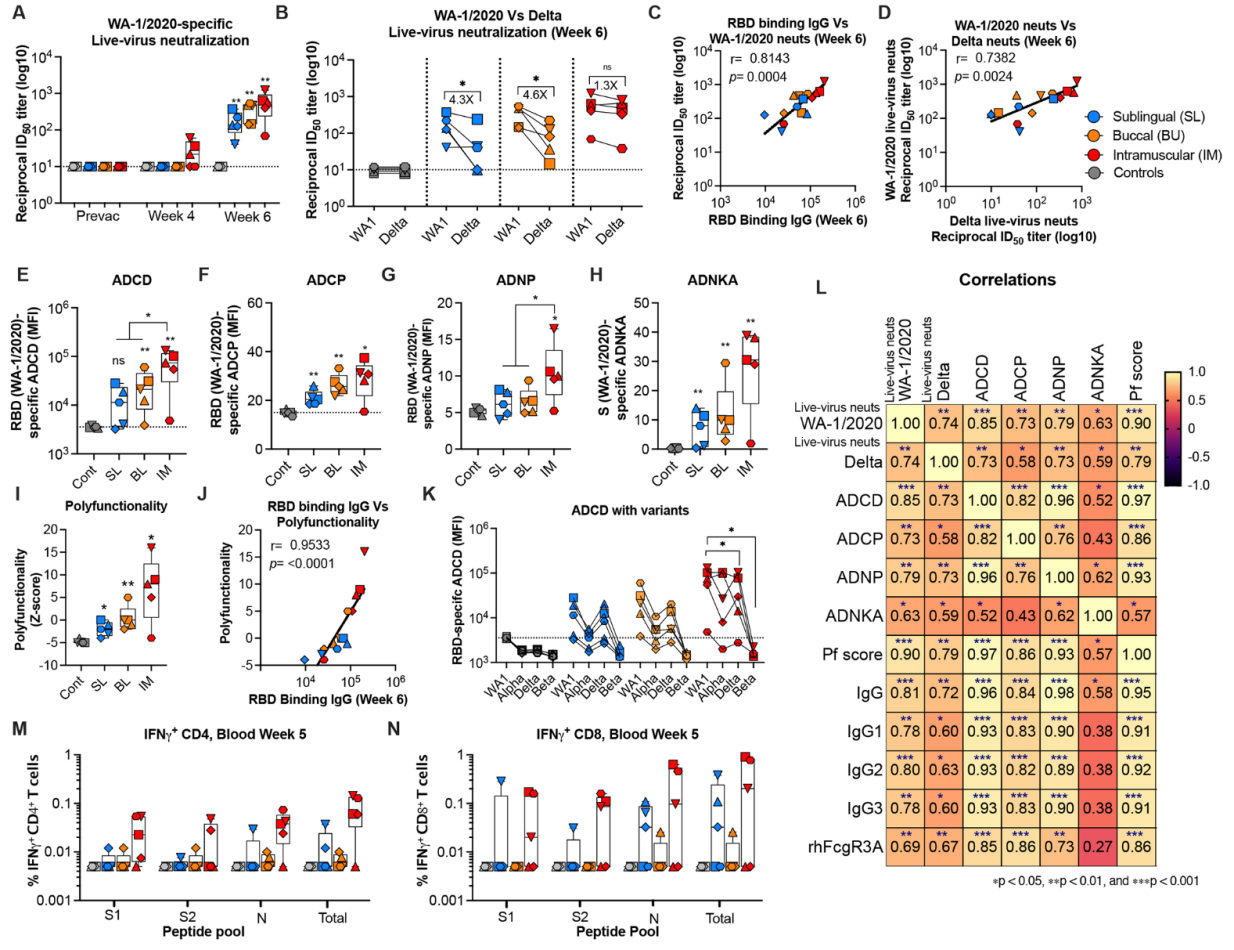
Functional antibody responses elicited following MVA/SdFCS-N immunization in rhesus macaques. (**A**) Sera collected at pre- (week 0) and post- (week 4 and 6) vaccination timepoints were used to assess live-virus WA-1/2020-specific neutralization activity. (**B**) Cross-reactive delta variant-specific cross-neutralization activity (week 6 only). Each sample was analyzed in duplicate. (**C-D**) Correlation analysis between RBD (WA-1/2020) binding titers and live-virus neutralization of WA-1/2020 (**C**), and between live-virus neutralization titers of SARS-CoV-2 WA-1/2020 and delta live-viruses (**D**). RBD (WA-1/2020)-specific antibody-dependent effector functions (2-weeks post-boost): ADCD (**E**), ADCP (**F**), ADNP (**G**), S (WA-1/2020)-specific ADNKA (**H**), Polyfunctionality score (Z-scores)(**I**). (**J**) Correlation analysis between RBD (WA-1/2020) binding titers and polyfunctionality. The Spearman rank test was used to perform correlation analysis. (**K**) Cross-binding ADCD functions (2-weeks post-boost) against RBD of variants. (**L**) Correlation matrix analysis between antibody-dependent functions (neutralization, ADCD, ADCP, ADNP, ADNKA, and polyfunctionality score) and RBD (WA-1/2020)-specific antibody isotypes, and FcγR3A binding in serum at 6 weeks (2 weeks post-boost). The color refers to *r* value scale (−1 to 1) shown on the right. The number in each cell indicates the actual r value and the stars represent *p*-values. (**M-N**) S1, S2 and N-specific CD4+ (**M**) and CD8+ (**N**) T cells in blood at week 5 (week 1 post-boost) after re-stimulation with a peptide pool. S1, S1 region of spike residues 1-685; S2, S2 region of spike residues 686-1273; N, nucleocapsid; Sum, total response (S1 + S2 + N). Data represent one independent experiment. Whisker plots show the maximum and minimum values. Dotted lines indicate functional assay limits of detection. Data are mean ± SEM. A two-sided Mann–Whitney U-test was used to compare between groups, *p < 0.05, **p < 0.01, and ***p < 0.001.

Vaccine-induced antibodies showed high titers of homologous (WA-1/2020-specific) neutralizing activity against the live virus at 2 weeks post with 50% neutralization titer reaching as high as 1228 (**Fig. 4A** **)**. In addition, low neutralizing activity was observed at 4 weeks post prime in 3 out of the 5 animals in only the IM group. Vaccine-induced antibodies also showed cross-reactive neutralizing activity against the delta VOC (**Fig. 4B** **)**. The cross-reactivity titer was comparable to homologous titers in the IM group (1.3-fold decrease) but had a significant decrease in both oral groups (4.3-fold in SL group, 4.6-fold in BU group), indicating significant qualitative differences in the antibodies generated via different routes of vaccination. The live virus specific neutralizing antibody titers were positively correlated with RBD binding IgG titer **(** **Fig. 4C** **)**. Moreover, the live-virus specific neutralizing antibody titers of WA-1/2020 positively correlated with delta virus neutralization titers **(** **Fig. 4D**). These data demonstrated that the vaccine-elicited antibodies were capable of neutralizing heterologous delta VOC and this was potentially mediated through their cross-reacting binding activity to RBD region.

Non-neutralizing antibody functions play an important role in providing protection against SARS-CoV-2 infection (*32*, *33*). Vaccine-induced antibody displayed antibody dependent complement deposition (ADCD), antibody dependent cellular phagocytosis (ADCP), antibody dependent neutrophil phagocytosis (ADNP), and antibody dependent NK cell activation (ADNKA) activities (**Fig. 4, E** to **H** **)**. These activities were generally higher in the IM group compared to the two oral groups with a significant difference for ADCD and ADNP activities. The vaccine-induced sera also showed poly-functional antibody responses with IM vaccinated animals having highest polyfunctional scores (Z-score) (**Fig. 4I**). The polyfunctionality (Z-score) positively correlation with RBD (WA-1/2020) binding IgG titers **(** **Fig. 4J**). Similar to binding antibody response, the ADCD and ADCP functions against tested VOCs were lower especially to delta and beta strains relative to WA-1/2020 **(** **Fig. 4K** **) (fig. S1H).**

The vaccine-induced neutralizing (WA-1/2020-specifc) antibody titers and Fc-mediated effector functions ADCD, ADCP and ADNP had a significant positive correlation with RBD (WA-1/2020)-specific Ig subclasses and FcγR3A (**Fig. 4L**). However, the ADNKA showed weak correlation with RBD-specific IgG and polyfunctional score (**Fig. 4L**). Overall, these results demonstrated that the MVA/SdFCS-N vaccine-induced poly-functional antibody response with IM vaccinated animals had the strongest neutralizing and non-neutralizing effector function activities.

One animal in the IM group, RLu18, showed unusually low binding IgG antibody response in serum, non-neutralizing antibody functions, and IgG antibody responses in mucosal secretions. To understand this further we looked at several immune parameters at baseline (pre vaccination) including total naïve and memory T and B cells, clinical chemistries, and complete blood counts (**fig. S2**). These analyses revealed the presence of a very high baseline proliferation of B cells and CD4 and CD8 T cells, high neutrophils and low lymphocytes compared to all other animals suggesting a heightened inflammatory state (**fig. S2, C** to **E**) which could have contributed to poor humoral immunity in this animal.

### MVA/SdFCS-N vaccine induced S and N-specific CD4 and CD8 T cell responses

To determine T cell responses in vaccinated and control animals, we stimulated peripheral blood mononuclear cells (PBMCs) with overlapping peptide pools specific to the S1, S2, and N and measured the frequency of antigen-specific memory T helper (TH) cell subsets and CD8 T cells using the intracellular cytokine staining (ICS) assay one week after the boost immunization (**Fig. 4, M and N** **)(figs. S, 3** and **4)**. The IM vaccination generated IFNγ+ CD4 and CD8 T cells responses that were targeted to S1, S2 and N proteins. These CD4 and CD8 T cells also expressed IL-2 and TNFα but did not express IL-4 or IL-17 suggesting a Th1 bias **(figs. S, 3** and **4)**. The BU and SL vaccinations also generated vaccine-specific CD4 and CD8 T cells in the blood and the magnitude of T cell response was comparable between IM and oral groups (**Fig. 4N**). Overall, these data demonstrated that inclusion of N in the vaccine may expand the CD4 and CD8 T cell breadth.

### SARS-CoV-2 delta exhibits enhanced replication kinetics in the upper airway compared to WA-1/2020 in macaques

**Fig. 5.**
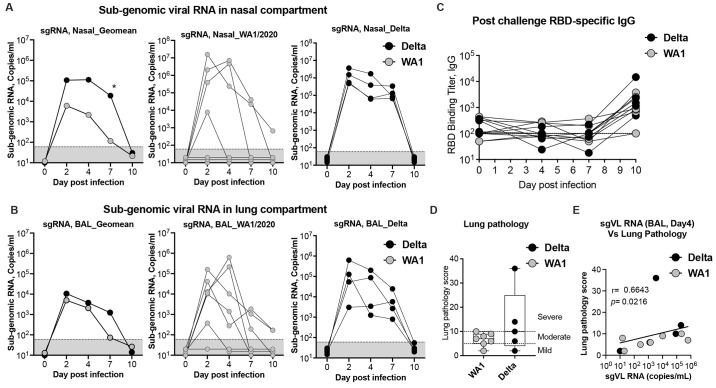
Viral replication kinetics of WA-1/2020 and delta (B.1.617.2) after intranasal (IN) and intratracheal (IT) infection in unvaccinated rhesus macaques. (**A-B**) Viral replication kinetics in the nasopharynx of the upper respiratory airways (upper panel) (**A**), and in bronchoalveolar lavage (BAL) fluid from the lower respiratory airways (lower panel) (**B**) in unvaccinated rhesus macaques following intranasal (IN) and intratracheal (IT) infection with WA-1/2020 (grey), and delta (B.1.617.2) (black) isolates. Replication was monitored from infection on day 0 until day 10 post-infection. Geometric mean titers (GMT) of sub-genomic RNA (sgRNA) copies/ml in log10 virus RNA copies per ml in nasopharyngeal and BAL samples (**A**), and from individual animal are shown. The limit of detection (<67 sgRNA copies/ml) is indicated in black dotted line. (**C**) RBD (WA-1/2020)-specific serum IgG responses in post-challenge serum. Each sample was tested in duplicates. (**D**) Lung pathology score at day 10 post-infection. (**E**) Correlation analysis between lung pathology scores and subgenomic viral loads (copies/mL). The Spearman rank test was used to perform correlation analysis. Each circle represents one animal. Comparison between groups was determined using a Mann–Whitney test. Error bars indicate the interquartile range with the median indicated by a horizontal line. Bars and columns show mean responses in each group ± SEM; Mann–Whitney test: ∗p < 0.05, and **p < 0.01. Dotted lines in viral loads reflect the limit of detection.

The SARS-CoV-2 delta VOC is more transmissible compared to other VOCs in humans and the mechanisms that contribute to its higher transmissibility compared to other VOCs are not clear. It is also not known how delta replicates in non-human primates (NHPs) and data from NHPs might provide important clues related to enhanced transmissibility of delta in humans. To understand this, we compared the viral replication kinetics in the upper and lower airways, lung pathology, and serum antibody responses between delta- and WA-1/2020-infected (historical, (*28*)) unvaccinated RMs. We measured sub-genomic RNA levels (envelope-specific) in nasopharynx (upper airway) and bronchoalveolar lavage (BAL)(lower airway) on days 2, 4, 7, and 10 (day of euthanasia) after intranasal (IN) and intratracheal (IT) infection (**Fig. 5A** **).** We observed significantly higher replication of delta for longer period in the upper airway compared to WA-1/2020. The virus replication peaked at day 2 in the nasopharyngeal compartment and the viral loads in delta infected animals were 17-fold higher compared to WA-1/2020 infected animals. This difference increased on day 4 (51-fold) and day 7 (164-fold), and by day 10 all animals became negative for sub genomic viral RNA (**Fig. 5A**). However, in the BAL, viral loads of delta infected animals were marginally higher compared to WA-1/2020 infected animals by 2.1-fold on day 2, 1.8-fold on day 4, and 17.1-fold on day 7, before dropping significantly by day 10 (**Fig. 5B**). Both delta and WA-1/2020 challenged animals developed a low titer of RBD binding IgG by day 10 but were not significantly different (**Fig. 5C**). In concordance with higher viral loads in the delta challenged RMs, lung pathology scores were also elevated but were not significantly higher than what was observed in WA-1/2020 infected animals (**Fig. 5D**). The day 4 p.i. BAL viral loads correlated directly with lung pathology scores (**Fig. 5E**). Overall, these data showed that delta virus replicates more efficiently in the upper airways for longer periods compared to WA-1/2020, and this longer persistence of high viral loads in the upper airway could explain its higher transmission and global predominance in humans.

### MVA/SdFCS-N vaccination protects against SARS-CoV-2 delta infection in macaques

**Fig. 6.**
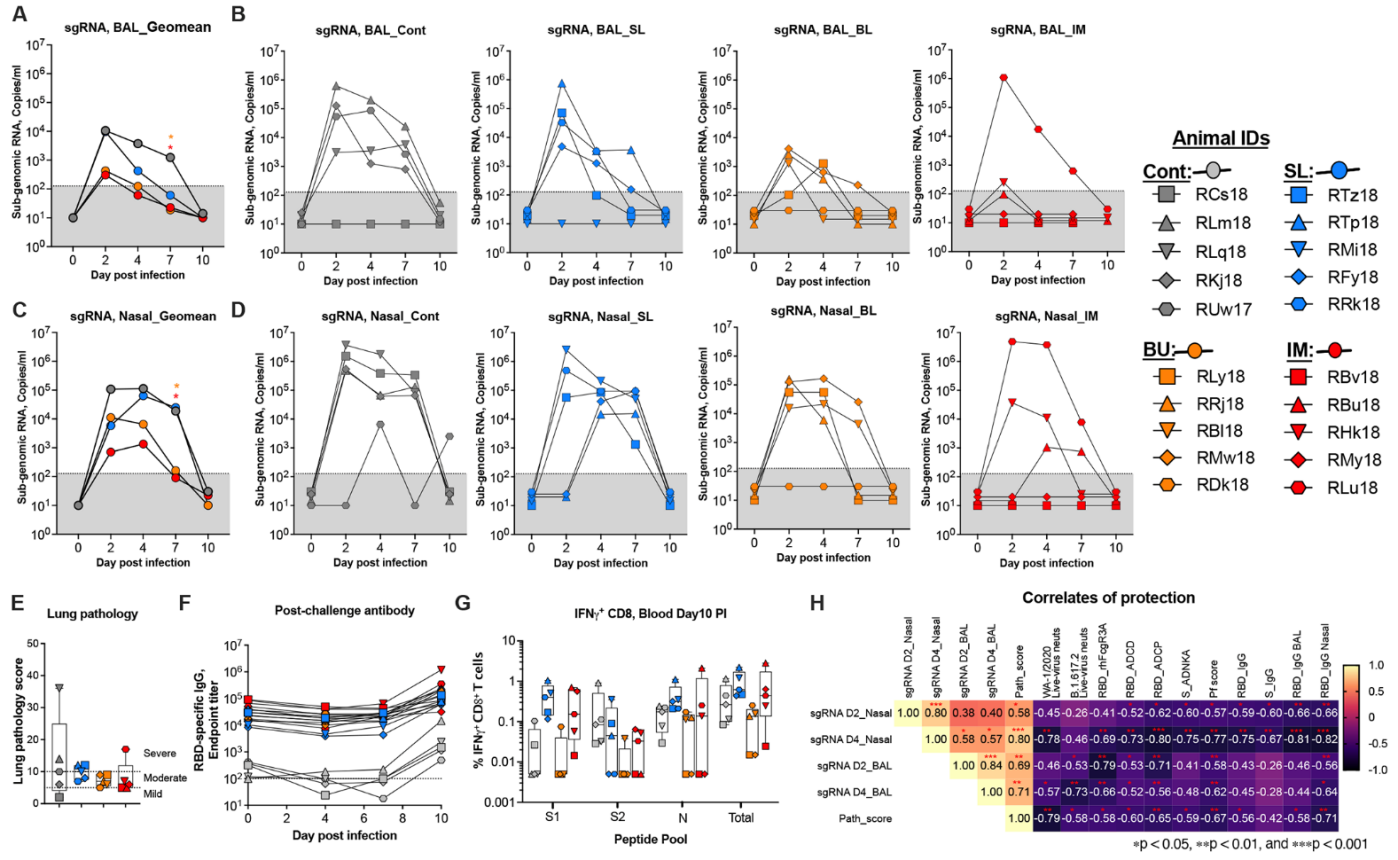
**Efficacy of MVA/SdFCS-N vaccination against upper and lower respiratory airway SARS-CoV-2 delta (B.1.617.2) viral replication. (A–E)** Rhesus macaques vaccinated as per **Fig. 3A** were challenged with SARS-CoV-2 delta (B.1.617.2) virus via intranasal (IN) and intratracheal (IT) route at week 8, and compared to MVA/Empty immunized controls. Quantified subgenomic RNA (sgRNA) of SARS-CoV-2 in bronchoalveolar lavage (BAL) fluid (**A-B**), and nasopharyngeal (NS) swab (**C-D**) collected on days 2, 4, 7 and 10 after challenge and presented as copies/ml. Geomean subgenomic viral loads (**A** and **C**) and individual animals subgenomic viral loads (**B** and **D**). (**E**) Lung pathology scores at day 10 post-infection. Hematoxylin and Eosin-stained lung sections were used to analyze tissue structure and cell infiltration. (**F**) RBD (WA-1/2020)-specific serum IgG responses in serum following challenge. Data represent one independent experiment. Each sample was tested in duplicates. (**G**) S1, S2 and N-specific CD8+ T cells in blood at day 10 post-infection after re-stimulation with a peptide pool. S1, S1 region of spike residues 1-685; S2, S2 region of spike residues 686-1273; N, nucleocapsid; Total, total response (S1 + S2 + N). (**H**) Correlation matrix analysis between sgRNA loads in nasopharyngeal (NS) swabs and bronchoalveolar lavage (BAL) fluids, versus 2 weeks post-boost (peak) timepoints RBD (WA-1/2020)-specific binding isotypes, and FcγR binding antibody-dependent functions (neutralization, ADCD, ADCP, ADNP, ADNKA, and polyfunctionality). The color refers to *r* value scale (−1 to 1) shown on the right. The number in each cell indicates the actual r value and the stars represent *p*-values. Whisker plots show the maximum and minimum values. Dotted lines in A to D, and F indicate the limits of detection for the assay. Data are mean ± SEM. A two-sided Mann–Whitney U-test was used to compare between groups, *p < 0.05 and **p < 0.01.

We challenged all vaccinated animals at week 8 (4 weeks post boost) to assess the vaccine efficacy (**Fig. 6**). Following challenge, the IM and BU vaccinated animals showed markedly lower virus replication compared to control animals both in the lower (**Fig. 6, A** and **B**) and upper (**Fig. 6, C** and **D**) airway starting from day 2, with a significant difference at day 7 (**Fig. 6, A** and **C**). In BAL (lower airway), the IM group animals showed profound viral control with 4 out of 5 animals showing viral loads below the level of detection until day 10 except a small blip of viral load (below 500 copies/ml) in two animals at day 2 (**Fig. 6B**). The exception was RLu18 which showed high virus replication on day 2 and controlled viral load gradually over time to below the level of detection by day 10. Notably, RLu18 was the only animal that showed high baseline T cell proliferation (**Fig. S2D**) and the only poor antibody responder animal in this group. This resulted in 35-, 62- and 55- fold lower viral loads on days 2, 4 and 7, respectively in the IM group compared to controls (**Fig. 6A**). Similar viral reduction was observed in the BU group except that the virus replication was more prominent in 3-4 animals on days 2 and 4 (**Fig. 6, A** and **B**). However, by day 7, 4 out of 5 animals controlled the virus to below the level of detection and by day 10 all animals were negative (**Fig. 6, A** and **B**). In contrast to IM and BU group animals, the SL group animals showed high viral loads at day 2 like control animals but showed a faster decline on days 4 and 7 (**Fig. 6, A** and **B**). Overall, these data showed that vaccination with MVA/SdFCS-N vaccine via IM and BU routes provided faster control of virus replication in the lower airway compared to controls and the SL route provided no protection.

In the nose, the viral control was more variable as has been observed with previous vaccines (*34*, *35*) however, the IM and BU group animals showed lower peak viremia and faster viral control at day 7 compared to the control group (**Fig. 6, C** and **D**). Two of the five IM vaccinated animals stayed negative for viral loads at all times tested post infection (**Fig. 6D**). The same two animals were also negative for viral loads throughout in BAL **(** **Fig. 6B** **)**. At day 4 post-challenge, the viral load had a trend for being lower in the IM and BU groups. By day 7, both the BU and IM groups had just 2 out of 5 RMs with detectable nasal viral loads, whose levels were significantly lower than in the control group (**Fig. 6, C** and **D**). By day 10, all vaccinated RMs were negative for viral loads, while one control animal still had detectable viral loads at 10^3^ copies/ml. The lung pathology was not significantly different between vaccinated and control groups. Most of the animals showed mild to moderate pathology (**Fig. 6E** **, Fig. S5** and **Table S1**). Three animals each in the control and SL groups, and one animal (RLu18) in the IM group showed severe pathology. Overall, these data showed that IM and BU routes of MVA/SdFCS-N vaccination of RMs provided substantial control of delta virus replication in both upper and lower airways.

To further understand the post infection anamnestic immune responses, we measured post-challenge RBD and N (WA-1/2020)-specific antibody responses in the serum on days 0, 4, 7, and 10 (**Fig. 6F** **) (fig. S6A**), and S and N specific T cell responses on day 10 (**Fig. 6G** **) (figs. S6, B** and **C**). In the vaccinated animals, the RBD binding titers remained stable through day 7 and increased 3-6 fold at day 10 in animals that showed high virus replication. The control animals had low RBD binding antibody on day 10. Similarly, a modest increases (not significant) in N binding IgG titers were observed on day 10 (**fig. S6A**). With respect to the T cell response in blood, both control and vaccinated animals showed low frequencies of IFNγ+ CD4 T cells that were directed against S1, S2 and N proteins (**fig. S6, B** and **C**). However, the magnitude of IFNγ+ CD8 T cell response was different between the groups. The control RMs showed low frequencies of IFNγ+ CD8 T cells that were mainly targeted to S2 and N (**fig. S6C**). In contrast, the IFNγ+ CD8 T cell response in the IM and SL vaccinated animals was mainly directed against S1 and N. In addition, the IFNγ+ CD8 T cell response in the BU group was mostly below the level of detection. Overall, we observed higher expansion of CD8 T cell response post infection compared to their respective post vaccination frequencies in animals that showed higher virus replication (**fig. S, 7** and **8)**.

We also measured S1, S2 and N-specific IFNγ+ CD4 and CD8 T cells in the lung draining hilar LN at day 10 post-infection (**fig. S9**). The vaccinated RMs showed a trend toward higher frequencies of IFNγ+ CD4 T cell response that was targeted to S1, S2 and N compared to control animals (**fig. S9A**). However, this trend was not observed for IFNγ+ CD8 T cell response, which was highly variable within and across different groups (**fig. S9B**). The IFNγ+ CD8 T cell response in the SL group showed consistently higher response suggesting a possible expansion toward relatively higher replicating virus among the three vaccinated groups. We observed a moderate but significant negative correlation between S1-specific IFNγ+ CD4 T cells in the hilar LN and day 2 viral loads in the nose (**fig. S9C**) and did not observe any other significant correlations between vaccine-induced CD4 or CD8 T cell response and protection. These data suggest a contribution of CD4 help in the lung in providing protection potentially through inducing better mucosal antibody response.

### Immune correlates for protection

For analyses of immune correlates of protection, we used multiple antibody functional activities elicited at the peak during vaccination (2 weeks post boost) (**Fig. 6H**). RBD-specific IgG in nasal secretions and serum ADCD and ADCP activities showed an inverse association with day 2 and day 4 viral loads in the nose and BAL. The ADNKA activity showed an inverse association with day 2 and day 4 viral loads in the nose but not in BAL. The live virus neutralizing activity showed an inverse correlation with day 4 viral loads in the nose and BAL but not with day 2 viral loads. The RBD-specific RhFcγRIIIA binding activity exhibited an inverse association with day 4 viral loads in the nose, day 2 and day 4 viral loads in BAL, and with lung pathology scores. In addition, antibody polyfunctional score showed an inverse association with multiple virological and pathological parameters including day 2 and day 4 viral loads in nose, day 2 and 4 viral loads in BAL and with lung pathological scores. The day 4 viral loads in BAL and nose showed a direct association with lung pathology score indicating higher virus replication resulting in higher lung pathology, as expected. With respect to contribution of T cells, we observed a moderate but significant inverse association between S1-specific IFNγ+ CD4 T cells in the hilar LN and day 2 viral loads in the nose (**fig. S9C**) and did not observe any other significant correlations between vaccine-induced CD4 or CD8 T cell response and protection. Overall, these data suggested that MVA/SdFCS-N vaccine induced multiple spike-specific antibody functions contributed to vaccine-mediated protection against heterologous SARS-CoV-2 infection. They also suggested that non-neutralizing effector functions played an important role in early virus control.

## DISCUSSION

mRNA and adenovirus-based vaccines robustly reduce COVID-19 incidence and severity, but their protection wanes with time and is less effective against VOCs compared to the wild-type Wuhan/Washington strain (*36*–*38*). COVID-19 vaccines that elicit broadly protective humoral and cellular immune responses are critical for enhanced protection against these emerging variants and mRNA vaccines achieve broad humoral immunity after 3 vaccinations (*39*, *40*). However, it is unclear how long the current vaccines will continue to be effective. Moreover, availability of multiple COVID-19 vaccines that elicit broadly protective humoral and cellular immune responses may help resolve the problem of getting safe and effective COVID vaccines to poor and middle-income countries to effectively cutdown the COVID-19 pandemic. Additionally, an effective vaccine should induce durable protective humoral and cellular immune responses in both the systemic and mucosal compartments against this mucosal-replicating virus. Here, to further improve and broaden the immunogenicity and efficacy of our MVA/S vaccine (*28*), we designed MVA/SdFCS-N vaccine that co-expressed two antigens with distinct immunologic goals. To induce strong neutralizing antibodies, we used prefusion-stabilized spike protein with its furin-cleavage site inactivated. We then added the highly conserved N gene to broaden the cellular responses to VOCs. Intramuscular delivery of MVA/SdFCS-N vaccine induced strong antibody responses with neutralizing and diverse non-neutralizing effector functions against the homologous WA-1/2020 strain and heterologous delta variant. The vaccine-induced immunity conferred protection against homologous and heterologous VOCs (beta and delta) in mice and macaques. The protection against beta variant was particularly good as this occurred despite low cross-neutralizing activity against this VOC. Collectively, these results revealed the potential of MVA/SdFCS-N vaccine to confer heterologous protection against future SARS-CoV-2 variants. We are yet to measure cross-reactivity of neutralizing antibody response induced by our MVA/SdFCS-N vaccine against Omicron. Based on recent data in humans and NHPs, three doses of mRNA vaccines are required to induce neutralizing antibody response against Omicron. Accordingly, we think a third MVA immunization might be required to induce a strong cross-reactive neutralizing activity against Omicron, however we predict a reasonable cross-reactivity of non-neutralizing effector functions and T cell responses that could contribute to protection.

The delta variant was the most dominant VOC across the world and was replaced by omicron recently, and there is a great need for understanding the mechanisms that leads to dominance of these newer SARS-CoV-2 VOCs. While the mechanisms related to greater transmissibility of delta and omicron VOCs are still being worked out, our results provide some important clues about greater transmissibility of delta VOC. In humans, delta infections result in higher viral loads in the unvaccinated as compared to alpha variant, with a difference that is more pronounced at >5 days post-symptom onset (*41*). The spike protein of delta also binds with higher affinity to human ACE2 receptor resulting in faster cell entry and increased ability to induce lung cell syncytia relative to wild-type (*12*, *42*). These results suggest that higher viral loads in delta infected individuals likely contribute to its increased transmissibility. However, in humans it is difficult to compare viral loads between variants given that many individuals with delta infections likely had pre-existing immunity to WA-1 via vaccination or prior natural infection and the infecting viral inoculum is inconsistent. Our results in NHPs also showed that delta infection resulted in higher viral loads with longer viral shedding when compared to WA-1/2020 infection. These results are consistent with and augment the less-controlled human data.

There is a great need for understanding the immune correlates for protection against SARS-CoV-2, especially against the emerging VOCs. Our study with MVA/S vaccine (*28*) and other studies with Moderna and Ad26.COV2.S vaccines in NHPs showed a robust neutralizing antibody (NAb) response contributing to protection in the lower and upper airway (*28*, *43*). In addition, a recent study suggested an important role for non-neutralizing antibody effector functions for protection in the upper airway (*33*, *44*). All these studies evaluated immune correlates for protection against homologous SARS-CoV-2 challenge and the immune correlates against heterologous SARS-CoV-2 challenge are still emerging. Our data against heterologous SARS-CoV-2 challenge in this study suggested a role for both neutralizing and non-neutralizing antibody responses in protection. Importantly, they also suggested that non-neutralizing effector functions play a more significant role than neutralizing antibody response especially at day 2 post infection in the upper airway. These results highlighted the important contribution of non-neutralizing effector functions in protection against VOCs by vaccines expressing WA-1 spike protein (*32*, *33*). Another study showed that there is a better cross reactivity of non-neutralizing effector functions against VOCs for total spike compared to RBD (*45*). Collectively, these results suggest that SARS-CoV-2 vaccines should use complete spike protein to induce a highly cross-reactive antibody response with diverse effector functions against VOCs.

With respect to the role of T cells in protection against SARS-CoV-2, a CD8-depletion study in NHPs vaccinated with Ad26.COV2.S vaccine showed that S specific CD8+ T cells could play a role in upper airway protection (*43*). Similarly, Ad5 expressing the N protein significantly reduces SARS-CoV-2 virus replication in mice and hamsters (*46*). Another study showed that the addition of N gene helps with control of virus replication in the brain of infected mice (*47*). Accordingly, we included N gene in our vaccine with the hope that a broader T cell response will enhance protection. However, we did not observe a significant association between vaccine induced T cell response in the blood and protection in our NHP study. It is possible that the neutralizing and non-neutralizing antibody effector functions could have masked the potential benefits of CD8 T cells in protection. In addition, the frequency of N specific CD8 T cells that we induced in our NHP study are markedly lower compared to the frequencies induced by Ad5 immunization in the above discussed mouse studies. Toward this, heterologous prime/boost approaches such as DNA prime/MVA boost and adenovirus vector prime/MVA boost can be effective to induce a robust CD4 and CD8 T cell as has been shown for HIV vaccines (*48*, *49*). In addition, the precise contribution of N-specific T cells in protection can only be addressed by comparing protection in MVA/SdFCS and MVA/SdFCS-N vaccinated macaques, which is outside the scope of this study.

The oral cavity is very rich in APCs and lymphoid tissue and is an attractive site for mucosal vaccination. In addition, oral vaccination induces greater antibody responses in the gastrointestinal tract compared to intramuscular vaccination (*50*). Consistently, oral vaccines have been licensed to use against viruses that transmit through fecal-oral route (*51*). However, most of the oral vaccination studies deliver the vaccines either topically in the mouth or intragastrically, and this limits antigen uptake across multi-layered oral mucosa. In contrast, in this study we used needle-free vaccination to deliver vaccine directly into oral mucosal tissue with the goal to bypass the mucosal barrier for antigen uptake. In addition, it was not known if needle-free vaccinations in the oral cavity could induce strong antibody response in the throat, nose and lung, the most important sites of SARS-CoV-2 replication. We had hypothesized that oral mucosal vaccination might provide superior mucosal immunity in these compartments, but instead found that IM vaccinations induce higher magnitude of antibodies in serum and mucosal secretions compared to oral needle-free vaccination in the mouth. In addition, oral vaccinations induced greater reduction in cross-reactive neutralizing antibody response against delta VOC compared to IM vaccination indicative of qualitative differences in antibodies elicited by immunization via the different routes. One possibility is that the vaccine administration sites process and present antigen in different ways, perhaps resulting in altered exposure of neutralizing and non-neutralizing antibody epitopes or altered migration of B cells. Notably, this may vary by antigen type, as we observed such differences with spike antibody titers but not with N-specific antibody titers.

One of the goals of our study was to compare immunogenicity and efficacy of SL and BU routes of immunization as our previous study used both sites simultaneously (*52*). SL and BU induced similar magnitude and functional quality humoral responses. Despite this, BU yielded significantly greater protection than SL in controlling delta virus replication. One possible explanation is that due to underlying differences in the tissue architecture of the buccal versus sublingual areas resulted in differential antigen uptake into draining lymph nodes. We previously showed that the buccal tissue contains higher proportion of BDCA-1+ conventional dendritic cells (cDCs) and dermal DCs compared to sublingual tissue (*52*). The presence of higher frequencies of antigen-presenting cells in the buccal tissue compared to sublingual mucosa could have resulted in their increased migration to draining lymph nodes leading to better antibody maturation in the BU group, perhaps accounting for the superior control of delta viral replication in this group. In summary, these results showed that improved MVA-based SARS-CoV-2 vaccine delivered intramuscularly elicits strong spike-specific antibody response with diverse functions and T-cell responses to spike and N, which contribute to protective immunity against homologous and heterologous SARS-CoV-2 variants. They also highlight the potential of MVA/dFCS-N vaccine as the initial or booster vaccine against future emerging variants.

## MATERIALS AND METHODS

### Study design

The study was designed with to evaluate the immunogenicity and efficacy of a modified vaccinia Ankara (MVA) virus vector-based SARS-CoV-2 vaccine co-expressing the furin-cleavage site inactivated spike and nucleocapsid (MVA/SdFCS-N) against heterologous SARS-CoV-2 challenge. We characterized the expression and confirmation of SdFCS using flow cytometry and Western blot techniques. We first immunized mice with MVA/SdFCS vaccine and showed that it induced markedly higher neutralizing antibody response against SARS-CoV-2 and protected against homologous and heterologous SARS-CoV-2 challenge. We then immunized rhesus macaques with MVA/SdFCS-N vaccine via intramuscular and oral (at buccal or sublingual sites) routes and challenged with delta VOC. We characterized the breadth and magnitude of humoral and cellular immune responses induced by the vaccine and measured associations between these and post infection viral loads to define immune correlates for protection against heterologous SARS-CoV-2 infection. We used 5 animals/group based on our previous experience in detecting immune responses and assessing protective efficacy in vaccine studies in macaques. Serum samples were collected to analyze neutralizing and non-neutralizing antibody functions. Swab and bronchoalveolar lavage fluid samples were collected to quantify mucosal antibody levels and viral loads in respiratory airways. In addition, we evaluated lung pathology at euthanasia using histology.

**Rhesus macaque study** – Twenty male Indian rhesus macaques (Macaca mulatta), 3-5 years old, were housed at Yerkes National Primate Research Center, Emory University in pairs in standard non-human primate cages and provided with both standard primate feed (Jumbo Monkey Diet 5037; Purina Mills, St. Louis, MO), fresh fruit, and enrichment daily, as well as free access to water. Immunizations, blood draws, and other sample collections were performed under anesthesia with ketamine (5-10 mg/kg) or telazol (3-5 mg/kg) by trained research and veterinary staff. Animals were randomly allocated into four groups, n=5 per group. Groups 1 to 3 received MVA/SdFCS-N vaccine via Sublingual (SL), Buccal (BU) and Intramuscular (IM) routes; Group 4 received MVA-empty vector (MVA/Empty) via IM route. Animals received 1x10^8^ PFU at weeks 0 and 4. The PharmaJet Tropis Needle-Free delivery system was used to deliver the vaccine for the SL and BU animals in 0.1ml volume, and a conventional 25-gauge needle was used to deliver the vaccine for IM and control groups in 1 ml volume. At week 8, macaques were challenged with a total of 1x10^5 PFUs of SARS-CoV-2 B.1.617.2 variant (delta VOC, CDC stock PP4P1; titered on Vero-TMPRSS2 cells). The integrity of the challenge delta virus (CDC stock PP4P1) was validated by confirming in vitro infectivity following viral passage, and by genomic sequencing to confirm the presence of all delta variant spike protein mutations. The virus was administered as 2 ml by the intratracheal (IT) route, and 1 ml by the intranasal (IN) route (0.5 ml in each nostril). Nasopharyngeal swabs and BAL samples were collected, stored immediately in viral transport media, and processed for viral RNA extraction on the same day. All vaccinated and non-vaccinated macaques were euthanized at day 10 after the SARS-CoV-2 challenge. Necropsy samples were collected (lung tissues) and stained with Hematoxylin and Eosin.

**Mouse study** - Specific-pathogen-free (SPF) 6–8-week-old female BALB/cJ and C57BL/6J mice were obtained from Jackson Laboratories (Wilmington, MA, USA) and housed in the animal facility at the Yerkes National Primate Research Center of Emory University, Atlanta, GA. All the rodents were maintained on a 12hr light/dark cycle at a temp/humidity of 68-79F/30 – 70% and animal rooms were monitored electronically through an automated system. All animals were fed a standard rodent chow (Purina Laboratory Rodent Diet 5001; Purina Mills, St. Louis, MO). Mice were immunized with 10^7 plaque-forming-units (PFU) of the MVA/S or MVA/SdFCS vaccine in the thigh muscle (dose split equally into each thigh) using a 25-gauge needle on weeks 0 and 4. 2-weeks after each immunization, blood samples were collected by facial vein puncture in BD Microtainer® Tubes for analyzing SARS-CoV-2 S (RBD)-specific serum antibody responses. In some experiments animals were challenged with either SARS-CoV-2 (MA10, WA-1/2020) virus or beta variant (B.1.351). MA10, WA-1/2020 challenge experiments were carried out at the University of North Carolina (UNC) at Chapel Hill. Beta variant (B.1.351) challenge experiments were conducted at Emory University. Briefly, mice were anesthetized using ketamine/xylazine and infected intranasally with 10^5 PFU of virus diluted in PBS (*30*). Clinical signs of disease (weight loss and body score) were monitored daily. The mice were euthanized by isoflurane overdose at indicated time points when samples for titer (caudal right lung lobe) were collected. Plaque assays and sub genomic Viral RNA were used to define lung viral titers as described previously (*53*).

### Ethics Statements

Mice and rhesus macaques were housed at either the Yerkes National Primate Research Center or UNC, and animal experiments were approved by the respective Institutional Animal Care and Use Committee (IACUC). Both facilities are AAALAC accredited and all animal experiments were carried out in accordance to USDA regulations and recommendations derived from the Guide for the Care and Use of Laboratory Animals.

### Cells and Viruses

HEK (Human Embryonic Kidney)-293T cells, DF-1 (Chicken embryo fibroblasts), and Vero cells were obtained from ATCC. The titer of MVA viruses was determined using DF-1 cells and SARS-CoV-2 viruses (2019-nCoV/USA_WA-1/2020, beta (B.1.351) and delta (B.1.617.2) using VeroE6 cells. VeroE6 cells and DF-1 cells were cultured in complete DMEM medium consisting of 1x DMEM (Corning Cellgro), 10% Fetal bovine serum (FBS), 25 mM HEPES Buffer (Corning Cellgro), 2 mM L-glutamine, 1mM sodium pyruvate, 1x Non-essential Amino Acids, and 1x antibiotics. Viral stocks were stored at -80°C until further use.

### Construction and characterization of MVA vaccines

MVA/S vaccines express recombinant full-length spike (amino acids 1-1273) with the K986P/V987 prefusion-stabilizing mutations; MVA/SdFCS additionally included the R682S/R685G modifications to inactivate the furin cleavage site. The full-length spike and the nucleocapsid (N) sequence of the SARS-CoV-2 Wuhan Hu-1 (WA-1/2020) strain was obtained from GenBank and codon optimized for vaccinia virus codon usage, synthesized commercially (GenScript, Piscataway, NJ) and subcloned in between *Xma*1 and *BamH*1 restriction sites of the plasmid transfer vector pLW-73 to transfer the inserts in between 2 essential genes I8R and G1L, under the control of an independent early/late vaccinia virus promoter (modified H5 [mH5]). Similarly, nucleocapsid subcloned in between XmaI and SaII restriction sites of the plasmid transfer vector pLAS-2 (provided by L. Wyatt, National Institutes of Health) to transfer the inserts into deletion II site of MVA under the control of an independent early/late vaccinia virus promoter (modified H5 [mH5]) (*54*). The recombinant MVAs were generated and confirmed by standard procedures. The integrity of the sequences was confirmed by PCR. Protein expression and cell surface expression by rMVAs was confirmed by infection of DF-1 cells followed by flow cytometry using human ACE2 (hACE2) binding and Spike specific antibodies. The nucleocapsid expression was confirmed by commercially available mAb antibody. Vaccine stocks were grown using DF-1 cells. Viral stocks were purified from lysates of infected DF-1 cells using a 36% sucrose cushion and titrated using DF-1 cells by counting PFU/ml. Absence of the wild-type MVA was confirmed by PCR using recombinant specific primers flanking the inserts.

#### Flow staining for SARS-CoV-2 Spike protein expression and ACE2 binding

Analysis of SARS-CoV-2 Spike protein expression on the MVA vaccine infected DF-1 cell surface and ACE2 binding was performed by flow cytometry. Briefly, DF-1 cells were infected with MVA/S, MVA/SdFCS or MVA/SdFCS-N at an MOI of 1 and stained after 36hrs of post-infection. Please refer to supplementary file for detailed methods.

#### Western Blotting

DF-1 cells were infected either with recombinant MVA/antigen(s), at an MOI of 1 for 36 hours. Infected cell lysates and supernatants were collected to perform Western blot analysis as described previously (*28*). Please refer to supplementary file for detailed methods.

#### RBD-His Protein expression and purification

The RBD (WA-1/2020)-His protein was produced in Amara laboratory by transfecting HEK293 cells using plasmids pCAGGS-RBD-His as described previously (*28*, *53*). Please refer to supplementary file for detailed methods.

#### Binding antibody responses using ELISA

SARS-CoV-2 S, RBD, N–specific IgG in serum were quantified by enzyme-linked immunosorbent assay (ELISA) as described previously (*28*). Please refer to supplementary file for detailed methods.

### Binding antibody multiplex assay (BAMA) and ELISA for mucosal antibodies

A customized BAMA was used to measure IgG and IgA antibodies in secretions and in serum specific for the RBD of SARS-CoV-2 WA-1/2020 Wuhan or delta variant and N protein as described previously (*55*). *Please refer to supplementary file for detailed methods.*

#### Live-virus neutralization

Live-virus SARS-CoV-2 neutralizing antibodies were assessed using a full-length mNeonGreen SARS-CoV-2 (2019-nCoV/USA_WA-1/2020), generated as previously described (*56*). Please refer to supplementary file for detailed methods.

#### *Antibody isotype, IgG* subclass, IgA, IgM, and FcγR binding of monkey sera

A Luminex assay was used to detect and quantify antigen-specific subclass, isotype and Fc-receptor (binding) function as described previously (*57*). Please refer to supplementary file for detailed methods.

#### *ADCP, ADNP and ADCD Assays* for monkey sera

Antibody-dependent cellular phagocytosis (ADCP), antibody-dependent neutrophil phagocytosis (ADNP) and antibody-dependent complement deposition (ADCD) were measured as previously described (*58*–*60*). Please refer to supplementary file for detailed methods.

### Antibody-dependent natural killer cell activation (ADNKA)

A modified version of a previously described plate bound antibody-dependent natural killer cell activation (ADNKA) assay was used (*61*). Please refer to supplementary file for detailed methods.

#### Cell processing

Post SARS-CoV-2 challenge, samples were processed and stained in BSL-3 facility. For macaques, PBMC from blood collected in sodium citrate CPT tubes were isolated using standard procedures. For processing lymph-node, lymph-node biopsies were dissociated using 70 μm cell strainer. The cell suspension was washed twice with R-10 media. Pelleted cells were suspended in 1ml R10 medium (RPMI(1X), 10% FBS) and stained as described in sections below.

#### Intracellular Cytokine Staining (ICS) assay

Functional responses of SARS-CoV-2 S1, S2, RBD, and N-specific CD8^+^ and CD4^+^ T cells in vaccinated animals were measured using peptide pools and an intracellular cytokine staining (ICS) assay as described previously (*28*, *48*, *53*). Overlapping peptides (13 or 17-mers overlapping by 10 amino acids) were obtained from BEI resources (NR-52402 for spike and NR-52419 for nucleocapsid) and different pools (S1, S2, RBD and N) were made. The S1 pool contained peptides mixed from 1-97, the S2 pool contained peptides mixed from 98-181, the RBD pool contained peptides 46-76 and the N pool contained 57 peptides. Each peptide was used at a 1 μg/ml concentration in the stimulation reaction. Please refer to supplementary file for detailed methods.

#### Histopathological examination

The animals were euthanized at the study end point, and a complete histopathologic examination was performed at necropsy as described previously (*28*, *53*). For histopathologic examination tissue were fixed in 10% neutral-buffered formalin for 24h at room temperature, paraffin-embedded, sectioned at 4 μm, and stained with hematoxylin and eosin (H&E). The H&E slides were examined by a board-certified veterinary pathologist. For each animal, all the lung lobes were used for analysis and affected microscopic fields were scored semiquantitatively as Grade 0 (None); Grade 1 (Mild, 0-5 score); Grade 2 (Moderate, 6-12 score) and Grade 3 (Severe, 13-18 score). Scoring was performed based as described previously (*28*, *53*). Briefly, scoring was performed based on these criteria: number of lung lobes affected, type 2 pneumocyte hyperplasia, alveolar septal thickening, fibrosis, perivascular cuffing, peribronchiolar hyperplasia, inflammatory infiltrates, hyaline membrane formation. An average lung lobe score was calculated by combining scores from each criterion. For animals with multiple affected lung lobes, each lung lobe was assessed individually and then the scores for each category were averaged. The total score was then determined for each animal. Digital images of H&E stained slides were captured at ×100 and ×200 magnification with an Olympus BX43 microscope equipped with a digital camera (DP27, Olympus) using Cellsens® Standard 2.3 digital imaging software (Olympus).

#### Viral RNA extraction and quantification

SARS-CoV-2 subgenomic RNA was quantified in nasopharyngeal swabs and broncho-alveolar lavages (BAL) as described previously (*28*, *53*). Quantitative reverse transcription PCR (qRT-PCR) was performed on the subgenomic mRNA transcript of the E gene (*62*). qPCR reactions were performed in duplicate using 200nM of each primer and 125nM of the probe. The limit of detection in this assay was about 128 copies per ml of VTM/BAL. To verify sample quality, the CDC RNase P p30 subunit qPCR was modified to account for rhesus macaque specific polymorphisms. The primer and probe sequences are RM-RPP30-F 5′-AGACTTGGACGTGCGAGCG-3′, RM-RPP30-R 5′- GAGCCGCTGTCTCCACAAGT-3′, and RPP30-Pr 5′-FAM-TTCTGACCTGAAGGCTCTGCGCG-BHQ1-3′ (*63*). Please refer to supplementary file for detailed methods.

### Quantification and statistical analysis

The difference between any two groups at a time point was measured either using a two-tailed nonparametric Mann–Whitney rank-sum test or unpaired parametric *t* test depending on the distribution of the data. Comparisons between different time points within a group used paired parametric *t* tests. A p-value of less than 0.05 was considered significant. The correlation analysis was performed using the Spearman rank test. GraphPad Prism version 8.4.3 (GraphPad Software) was used to perform data analysis and statistics.
