# Supplementary material for: A modified vaccinia Ankara vaccine expressing spike and nucleocapsid protects rhesus macaques against SARS-CoV-2 delta infection
Source: Sci Immunol. 2022 Mar 31:eabo0226. doi: 10.1126/sciimmunol.abo0226 (PMC8995033; doi:10.1126/sciimmunol.abo0226)
Supplement: Supplementary file 1 — Materials and Methods Figs. S1 to S9 Table S1 [file sciimmunol.abo0226_sm.pdf]

Science Immunology
AAAS

Supplementary Materials for

A modified vaccinia ankara vaccine expressing spike and nucleocapsid protects rhesus macaques against SARS-CoV-2 delta infection

Nanda Kishore Routhu *et al.*

Corresponding author: Rama Rao Amara, ramara@emory.edu

DOI: 10.1126/sciimmunol.abo0226

The PDF file includes:

Materials and Methods
Figs. S1 to S9
Table S1

Other Supplementary Material for this manuscript includes the following:

Data file S1

A modified vaccinia ankara vaccine expressing spike and nucleocapsid protects rhesus macaques against SARS-CoV-2 delta infection

Nanda Kishore Routhu[1,2,†], Sailaja Gangadhara[1,2,†], Lilin Lai[1], Gardner Meredith Elizabeth Davis[1], Katharine Floyd[1], Ayalnesh Shiferaw[1,2], Yannic C Bartsch[3], Stephanie Fischinger[3], Georges Khoury[1], Sheikh Abdul Rahman[1,2], Samuel David Stampfer[1,2], Alexandra Schaefer[6], Sherrie M. Jean[4], Chelsea Wallace[4], Rachelle L. Stammen[4], Jennifer Wood[4], Cohen Joyce[4], Tamas Nagy[5], Matthew S. Parsons[1,6], Lisa Gralinski[7], Pamela A. Kozlowski[8], Galit Alter[3], Mehul S. Suthar[1,9] and Rama Rao Amara[1,2,*]

[1]Division of Microbiology and Immunology, Emory Vaccine Center, Yerkes National Primate Research Center, Emory University, Atlanta, Georgia 30329, USA.
[2]Department of Microbiology and Immunology, Emory School of Medicine, Emory University, Atlanta, Georgia 30322, USA.
[3]Ragon Institute of MGH, MIT and Harvard, Cambridge, Massachusetts 02139, USA.
[4]Division of Animal Resources, Yerkes National Primate Research Center, Emory University, Atlanta, Georgia 30329, USA.
[5]College of Veterinary Medicine, University of Georgia, Athens, Georgia 30602, USA.
[6]Department of Pathology and Laboratory Medicine, Emory University School of Medicine, Atlanta, GA, USA.
[7]Department of Epidemiology, University of North Carolina, Chapel Hill, North Carolina 27516, USA
[8]Department of Microbiology, Immunology, and Parasitology, Louisiana State University Health Sciences Center, New Orleans, Louisiana 70112, USA.
[9]Department of Pediatrics, Division of Infectious Diseases, Emory University School of Medicine, Atlanta, GA 30322, USA.

Footnotes

[†]Contributed equally to this work

[*]**Correspondence:** Correspondence should be addressed to Dr. Rama Amara. Phone: (404) 727-8765; FAX: (404) 727-7768; E-mail: ramara@emory.edu

Supplementary materials

Detailed materials and methods

Fig. S1. Cross-reactive antibody responses in the systemic and mucosal compartment elicited following MVA/SdFCS-N immunization in rhesus macaques.

Fig. S2. High baseline proliferating CD4+ and CD8+ T cells, high neutrophils and low lymphocytes observed in PBMCs of RLu18 animal at prevaccination timepoint.

Fig. S3. Gating strategy for intracellular staining (ICS) assay in flow analysis.

Fig. S4. The MVA/SdFCS-N vaccination induced T cell responses in the blood of NHPs.

Fig. S5. Post-challenge lung pathology in rhesus macaques.

Fig. S6. Nucleocapsid-specific antibody responses and T cell responses in the blood induced in NHPs following delta virus challenge.

Fig. S7. CD4+ T cell expansion following SARS-CoV-2 delta challenge, compared to post-vaccination (Week 5, 1-week after boost) responses in the blood of NHPs.

Fig. S8. CD8+ T cell expansion following SARS-CoV-2 delta challenge, compared to post-vaccination (Week 5, 1-week after boost) responses in the blood of NHPs.

Fig. S9. Spike- and N-specific CD4+ and CD8+ T cell responses in the hilar lymph node following SARS-CoV-2 delta challenge in macaques.

Table S1. Lung pathology scores of individual animals at necropsy (day 10 post infection).

Data file S1: Raw Data file

Materials and Methods

Flow staining for SARS-CoV-2 Spike protein expression and ACE2 binding

DF-1 cells were infected with MVA/S, MVA/SdFCS or MVA/SdFCS-N at an MOI of 1 and stained after 36hrs of post-infection. MVA/S infected cells were harvested and stained with live-dead dye and anti-SARS-CoV-2 RBD polyclonal antibody (#Cat 40592-T62, SinoBiological), followed by donkey anti-rabbit IgG coupled to PE (#Cat 406421, BioLegend). Cells were then fixed and permeabilized with Cytofix/cytoperm (BD Pharmingen), intracellularly stained with anti SARS-CoV-2 nucleocapsid (N) mouse monoclonal antibody (#Cat 40143-MM05, SinoBiological), followed by goat anti-mouse IgG coupled to APC, and for MVA using mouse monoclonal antibody (mAb) anti-vaccinia virus E3L antibody (#Cat NR-4547, BEI Resources) coupled to PacBlue. For detection of human ACE2 binding to surface expressed spike on MVA/S, MVA/SdFCS and MVA/SdFCS-N infected DF-1 cells, MVA/antigen-infected cells were incubated with biotinylated human ACE2 protein at 1:500 dilution (#Cat 10108-H08H-B, SinoBiological**)** followed by streptavidin-PE. Cells were then stained intracellularly for MVA as described above.

Western Blotting

DF-1 cells were infected either with recombinant MVA/antigen(s), at an MOI of 1 for 36 h. Infected cells were lysed in ice-cold RIPA buffer and supernatants were collected. Lysates were kept on ice for 10 min, centrifuged, and resolved by SDS PAGE using precast 4–15% SDS polyacrylamide gels (BioRad). Proteins were transferred to a nitrocellulose membrane, blocked with 1% casein blocker overnight (Cat# 1610782 BioRad), and incubated for 1 h at room temperature with primary antibody at 1:2500 in blocking buffer. MVA/S, MVA/SdFCS and MVA/SdFCS-N blots utilized mouse monoclonal anti-SARS-CoV-2 spike antibody (#Cat GTX632604, GeneTex) to detect spike, and MVA/SdFCS-N blots also used anti nucleocapsid mouse mAb as described earlier to detect nucleocapsid. The membrane was washed in PBS containing Tween-20 (0.05%) and incubated for 1 h with horseradish peroxidase-conjugated anti-mouse or anti-rabbit secondary antibody diluted at 1:20,000. The membranes were washed, and proteins were visualized using the ECL select chemiluminescence substrate (#Cat RPN2235, GEhealthcare).

RBD-His Protein expression and purification

The RBD (WA-1/2020)-His protein was produced in Amara laboratory by transfecting HEK293 cells using plasmids pCAGGS-RBD-His as described previously (*1, 2*). Briefly, RBD-6x-His (Cat# NR-52309, BEI resources) expressed in HEK 293F cells was purified using Ni-NTA resin (ThermoFisher, cat.no. 88221), and a Superdex 200 10/300 GL (GE Healthcare) column on an Akta^{TM}Pure (GE Healthcare) system. The protein concentration was estimated using the BCA Protein Assay Kit (Pierce) and quality was assessed by SDS PAGE and Western blot.

Binding antibody responses using ELISA

SARS-CoV-2 S, RBD, N–specific IgG in serum were quantified by enzyme-linked immunosorbent assay (ELISA) as described previously (*1*). Briefly, recombinant SARS-CoV-2 proteins,RBD, S (#40589-V08H4, Sino Biological Inc) and Nucleocapsid (# NUN-C5227, Acro Biosystems) coated *Nunc* Maxisorp ELISA plates were used to assess endpoint titers of antigen-specific IgG antibody in serum of vaccinated animals. Mouse IgG and monkey IgG antibodies were detected using HRP-conjugated goat anti-mouse IgG and goat anti-monkey IgG secondary antibodies, respectively. ELISA endpoint titers were defined as the highest reciprocal serum dilution that yielded an absorbance >2-fold over background values.

Binding antibody multiplex assay (BAMA) and ELISA for mucosal antibodies

A customized BAMA was used to measure IgG and IgA antibodies in secretions and in serum specific for the RBD of SARS-CoV-2 WA-1/2020 Wuhan or delta variant and N protein as described previously (*3*). The standards were calibrated using anti-RBD and anti-N humanized IgG monoclonal antibodies and consisted of heat-inactivated pooled serum (for IgG assays) or IgG-depleted serum (for IgA assays) from SARS-CoV-2 infected humans. Antibody concentrations were calculated from standard curves and subsequently normalized relative to the total IgG or IgA concentration in the sample. Total IgG and IgA were measured by ELISA as described (*4*) using using goat anti-monkey IgG (AlphaDiagnostics) or clone IgA5-3B mouse anti-monkey IgA (Bio-Rad) to coat plates, rhesus IgG (Rockland) or rhesus IgA (NHP Reagent Program) as standards, and the above biotinylated antibodies, avidin-peroxidase and TMB (SouthernBiotech) to develop plates.

Live-virus neutralization

Live-virus SARS-CoV-2 neutralizing antibodies were assessed using a full-length mNeonGreen SARS-CoV-2 (2019-nCoV/USA_WA1/2020), generated as previously described (*5*). SARS-CoV-2 (B.1.617.2, delta variant) was produced and tittered in Vero-TMPRSS2 cells. Sera were incubated at 56°C for 30 min and manually diluted in duplicate in serum-free Dulbecco's Modified Eagle Medium (DMEM) and incubated with 750-1000 focus-forming units (FFUs) of infectious clone-derived SARS-CoV-2-mNG virus (*5*) at 37° C for 1 hour. The virus/serum mixture was added to VeroE6 cell (#CRL-1586, C1008, ATCC) monolayers, seeded in 96-well blackout plates, and incubated at 37° C for 1 hour. Post incubation, the inoculum was removed and replaced with pre-warmed complete DMEM containing 0.85% methylcellulose. Plates were incubated at 37° C for 24 hours. After 24 hours, the methylcellulose overlay was removed, cells were washed three times with phosphate-buffered saline (PBS) and fixed with 2% paraformaldehyde (PFA) in PBS for 30 minutes at room temperature. PFA was removed and fixed cells were washed twice with PBS. Foci were visualized using an ELISPOT reader (CTL ImmunoSpot S6 Universal Analyzer) under a FITC channel and enumerated using Viridot (*6*). Neutralization titers were calculated as follows: 1 - ratio of the (mean number of foci in the presence of sera: foci at the highest dilution of respective sera sample). Each specimen was tested in two independent assays performed at different times. The focus-reduction neutralization mNeonGreen live-virus 50% titers (FRNT-mNG_{50}) were interpolated using a 4-parameter nonlinear regression in GraphPad Prism 8.4.3. Samples that did not neutralize at the limit of detection at 50% were plotted at 10 for use in geometric mean calculations.

***Antibody isotype, IgG* subclass, IgA, IgM, and FcγR binding of monkey sera**

A Luminex assay was used to detect and quantify antigen-specific subclass, isotype and Fc-receptor (binding) function as described previously (*7*). Immune complexes were formed by mixing appropriately diluted plasma (1:100 for IgG1, IgG2, IgG3, IgG4, IgA, IgM, and 1:1000 for FcγRs) to antigen-coupled beads and incubating the complexes overnight at 4˚C. Murine secondary antibodies for each antibody isotype or subclass were used to detect antigen-specific antibody titer and probed with a PE-conjugated tertiary anti-mouse antibodies (Invitrogen). For FcγRs, biotinylated FcγRs were labeled with streptavidin-PE before addition to immune complexes. Fluorescence was measured with an iQue (Intellicyt) and analyzed using Forecyt software. Data is reported as median fluorescence intensity (MFI).

ADCP, ADNP and ADCD Assays for monkey sera

Antibody-dependent cellular phagocytosis (ADCP), antibody-dependent neutrophil phagocytosis (ADNP) and antibody-dependent complement deposition (ADCD) were measured as previously described (*8-10*). Antigen-coupled beads were incubated with appropriately diluted plasma (ADCP 1:200, ADNP 1:50, ADCD 1:10) for 2 hours at 37˚C to form immune complexes. For ADCP, 2.5x10^4 THP-1s cells were added and incubated for 16 hours at 37˚C. For ADNP, leukocytes were isolated from fresh peripheral whole blood and added to immune complexes at 5x10^4 cells/well and incubated for 1 hour at 37˚C. Neutrophils were detected using anti-human CD66b Pacific-Blue. For ADCD, lyophilized guinea pig complement was resuspended, diluted in gelatin veronal buffer with calcium and magnesium (GVB++, Boston BioProducts) and added to immune complexes. The deposition of C3 was detected using an anti-C3 FITC antibody. All functional assays were acquired with an iQue (Intellicyt) and analyzed using Forecyt software. For ADCP, events were gated on singlets and fluorescent cells. For ADNP, bead-positive neutrophils were defined as CD66b positive, fluorescent cells. For both ADCP and ADNP, a phagocytic score was defined as (percentage of bead-positive cells) x (MFI of bead-positive cells) divided by 10000. For ADCD, data was reported as median fluorescence of C3 deposition (MFI).

Antibody-dependent natural killer cell activation (ADNKA)

A modified version of a previously described plate bound antibody-dependent natural killer cell activation (ADNKA) assay was used (*11*). Wells of a 96 well ELISA plate were coated with 6 μg/ml SARS-CoV-2 spike antigen (2019-nCoV, Sino Biological) in PBS overnight at 4˚C. Nonspecific binding sites were blocked with 5% milk and 4% whey in blotting-grade blocker (BioRad) for 1 hour at RT and 1:100 diluted sera samples added afterwards. PBMCs were isolated from naïve macaques using sodium heparin CPT tubes. Isolated PBMCs were mixed with anti-human CD107a APC-H7 (clone H4A3, BD) in the presence of Brefeldin A and Monensin, and immediately added to the prepared ELISA plate at 5x10^5 cells/well. After 5 hours incubation at 37˚C, cells were stained with anti-CD14 BV605 (clone M5E2), anti-CD8 PerCP-Cy5.5 (clone SK1), anti-NKG2a PE (clone Z199), anti-CD3 BUV395 (clone SP34-2) and Live/Dead Aqua Viability Dye (Life Technologies) in PBS containing 1 mM EDTA for 30 min at RT in the dark. Cells were fixed and acquired. The relative expression of CD107a on LiveCD3-CD14-CD8+NKG2a+ single NK cells was reported.

Cell processing

Post SARS-CoV-2 challenge, samples were processed and stained in BSL-3 facility. For macaques, PBMC from blood collected in sodium citrate CPT tubes were isolated using standard procedures. For processing lymph-node, lymph-node biopsies were dissociated using 70 μm cell strainer. The cell suspension was washed twice with R-10 media. Pelleted cells were suspended in 1ml R10 medium (RPMI(1X), 10% FBS) and stained as described in sections below.

Intracellular Cytokine Staining (ICS) assay

Functional responses of SARS-CoV-2 S1, S2, RBD, and N-specific $CD8^+$ and $CD4^+$ T cells in vaccinated animals were measured using peptide pools and an intracellular cytokine staining (ICS) assay as described previously (*1, 2, 12*). Overlapping peptides (13 or 17-mers overlapping by 10 amino acids) were obtained from BEI resources (NR-52402 for spike and NR-52419 for nucleocapsid) and different pools (S1, S2, RBD and N) were made. The S1 pool contained peptides mixed from 1-97, the S2 pool contained peptides mixed from 98-181, the RBD pool contained peptides 46-76 and the N pool contained 57 peptides. Each peptide was used at a 1 μg/ml concentration in the stimulation reaction. Two million cells suspended in 0.2 ml of RPMI 1640 medium with 10% FBS were stimulated with 1 μg/ml CD28, 1 μg/ml CD49d co-stimulatory

antibodies, and different peptide pools. These stimulated cells were incubated at 37°C in a 5% CO_2 conditioned incubator. After 2 hrs of incubation, 1 µl Golgi-plug and 1 µl Golgi-stop/ml were added and samples were incubated for 4 more hours. After a total 6 hours of incubation, cells were transferred to 4°C overnight and stained the next day. Cells were washed once with FACS wash (1X PBS, 2% FBS and 0.05% sodium azide) and surface stained with Live/Dead-APC-Cy7, anti-CD3, anti-CD4 and anti-CD8, each conjugated to a different fluorochrome for 30 minutes at RT. The stained cells were washed once with FACS wash and permeabilized with 0.2 ml of Cytofix/Cytoperm for 30 minutes at 4°C. Cells were washed once with perm wash and incubated with anti-cytokine (IFNγ, TNFα, IL-2, IL-4, and IL-17) antibodies for 30 minutes at 4°C. Finally, the samples were washed once with perm wash and once with FACS wash and fixed in 4% paraformaldehyde for 20 minutes before acquisition on a BD LSR Fortessa flow cytometer. Data was analyzed using FlowJo software.

Viral RNA extraction and quantification

SARS-CoV-2 subgenomic RNA was quantified in nasopharyngeal swabs, and broncho-alveolar lavages (BAL) as described previously (*1, 2*). Quantitative reverse transcription PCR (qRT-PCR) was performed on the subgenomic mRNA transcript of the E gene (*13*). Swabs were placed in 1ml of Viral Transport Medium (#Cat VR2019-1L, VTM Labscoop). Viral RNA was extracted from NP swabs, and BAL on fresh specimens using the QiaAmp Viral RNA mini kit according to the manufacturer's protocol. Quantitative reverse transcription PCR (qRT-PCR) was performed on the subgenomic mRNA transcript of the E gene (*13*) using primer and probe sequences for are SGMRNA-E-F: 5'-CGATCTCTTGTAGATCTGTTCTC-3', SGMRNA-E-R: 5'-ATATTGCAGCAGTACGCACACA-3', and SGMRNA-E-Pr: 5'-FAM-ACACTAGCCATCCTTACTGCGCTTCG-3'. qPCR reactions were performed in duplicate with the Thermo-Fisher 1-Step Fast virus mastermix using the manufacturer's cycling conditions, 200nM of each primer, and 125nM of the probe. The limit of detection in this assay was about 128 copies per ml of VTM/BAL depending on the volume of extracted RNA available for each assay. To verify sample quality, the CDC RNase P p30 subunit qPCR was modified to account for rhesus macaque specific polymorphisms. The primer and probe sequences are RM-RPP30-F 5'-AGACTTGGACGTGCGAGCG-3', RM-RPP30-R 5'- GAGCCGCTGTCTCCACAAGT-3', and RPP30-Pr 5'-FAM-TTCTGACCTGAAGGCTCTGCGCG-BHQ1-3' (*14*). A single well from each extraction was run as described above to verify RNA integrity and sample quality via detectable and consistent cycle threshold values (Ct between 25-32).

Supplementary Figures

Figure S1

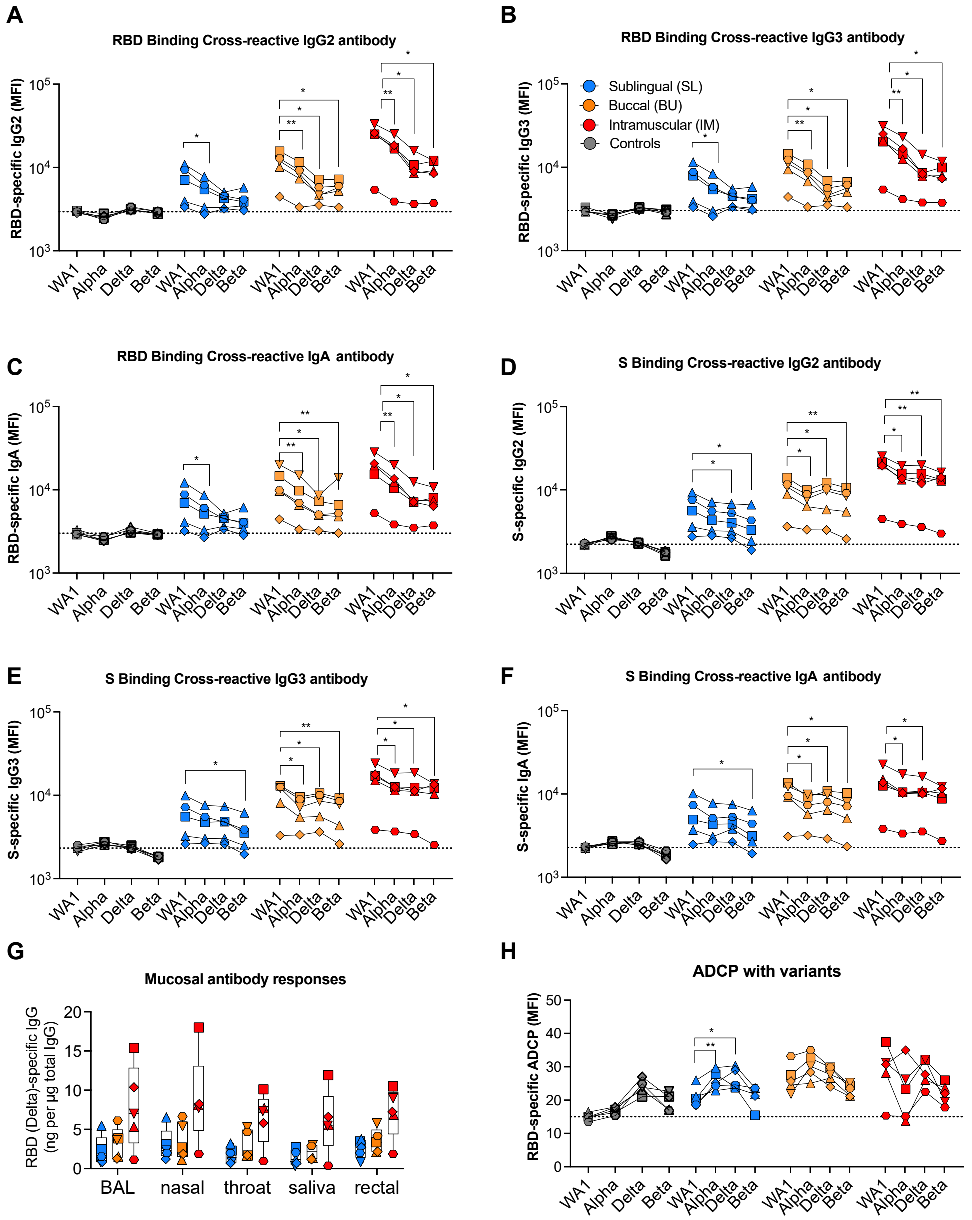

Figure S1. Cross-reactive antibody responses in the systemic and mucosal compartment elicited following MVA/SdFCS-N immunization in rhesus macaques. Serum cross-reactive RBD- (**A** to **C**) and S- (**D** to **F**) specific Ig subclass and IgA antibody responses elicited in RMs at two weeks after boost. Dotted lines indicate functional assay limits of detection. (**G**) RBD delta (B.1.617.2)-specific cross-binding IgG antibody in BAL, nasal, throat, saliva and rectal mucosal compartments, respectively. (**H**) RBD-specific ADCP functions. Data are mean ± SEM. A two-sided Mann–Whitney U-test was used to compare between groups, *p < 0.05, and **p < 0.01.

Figure S2

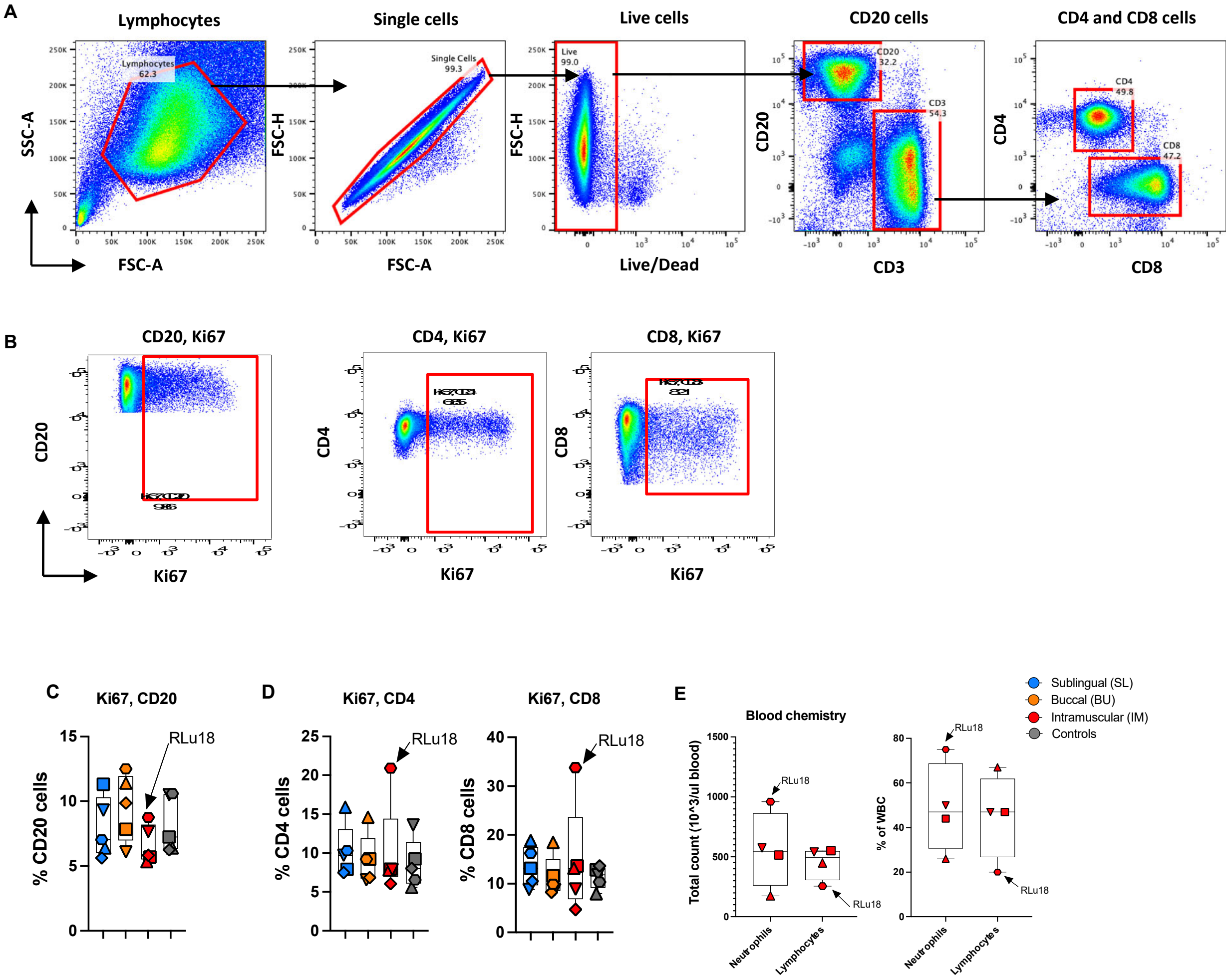

Figure S2. High baseline proliferating CD4 and CD8 T cells, high neutrophils and low lymphocytes observed in PBMCs of RLu18 animal at prevaccination timepoint. (A-B) Gating strategy used to define and quantify B and T cell subsets in PBMCs of NHPs. (A) Total lymphocytes (FSC-A and SSC-A) and singlets (FSC-A and FSC-H) were gated using scatter. Live cells were selected using live/dead marker. Total B cells were separated by CD3- and CD20+ gating. The T cells (CD3+) were then separated into CD4 versus CD8 co-receptor subsets. (B) The proliferating B and T cells were gated on Ki-67^{+} marker. (C) Frequency of proliferating (Ki67^{+}) B cell populations. (D) Frequency of proliferating (Ki67^{+}) CD4 and CD8 T cell populations. The quantifications of proliferating lymphocytes was carried out using PBMCs from the NHPs (n=20) that received no treatment or any immunizations. (E) Blood chemistry data showing absolute count (left) and % of white blood cells (right) of neutrophils and lymphocytes (missing blood chemistry data for one macaque). RLu18 9 (poor responder) is identified with arrow.

Figure S3

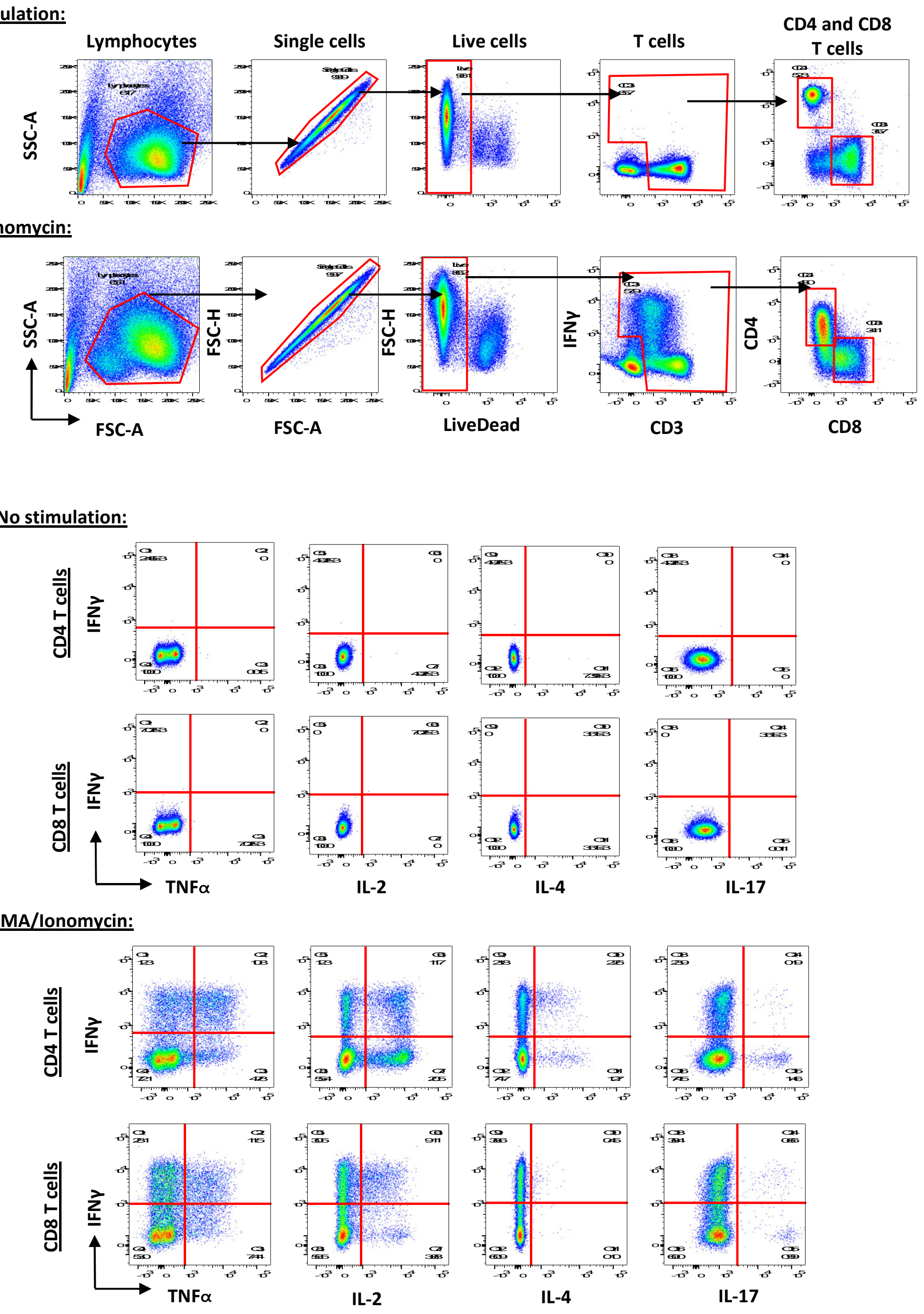

Figure S3. Gating strategy for intracellular staining (ICS) assay in flow analysis. No stimulations (Upper panel and lower left panel) and PMA/ionomycin (Lower panel and lower right panel). Total lymphocytes (FSC-A and SSC-A) and singlets (FSC-A and FSC-H) were gated using scatter. Live cells were selected using live/dead marker and followed CD3 and IFNγ expression was used to gate T cells. The T cells were then separated into CD4 versus CD8 co-receptor subsets. For CD4 and CD8 subset, cytokines. Phorbol myristate acetate (PMA) and Ionomycin positive controls.

Figure S4

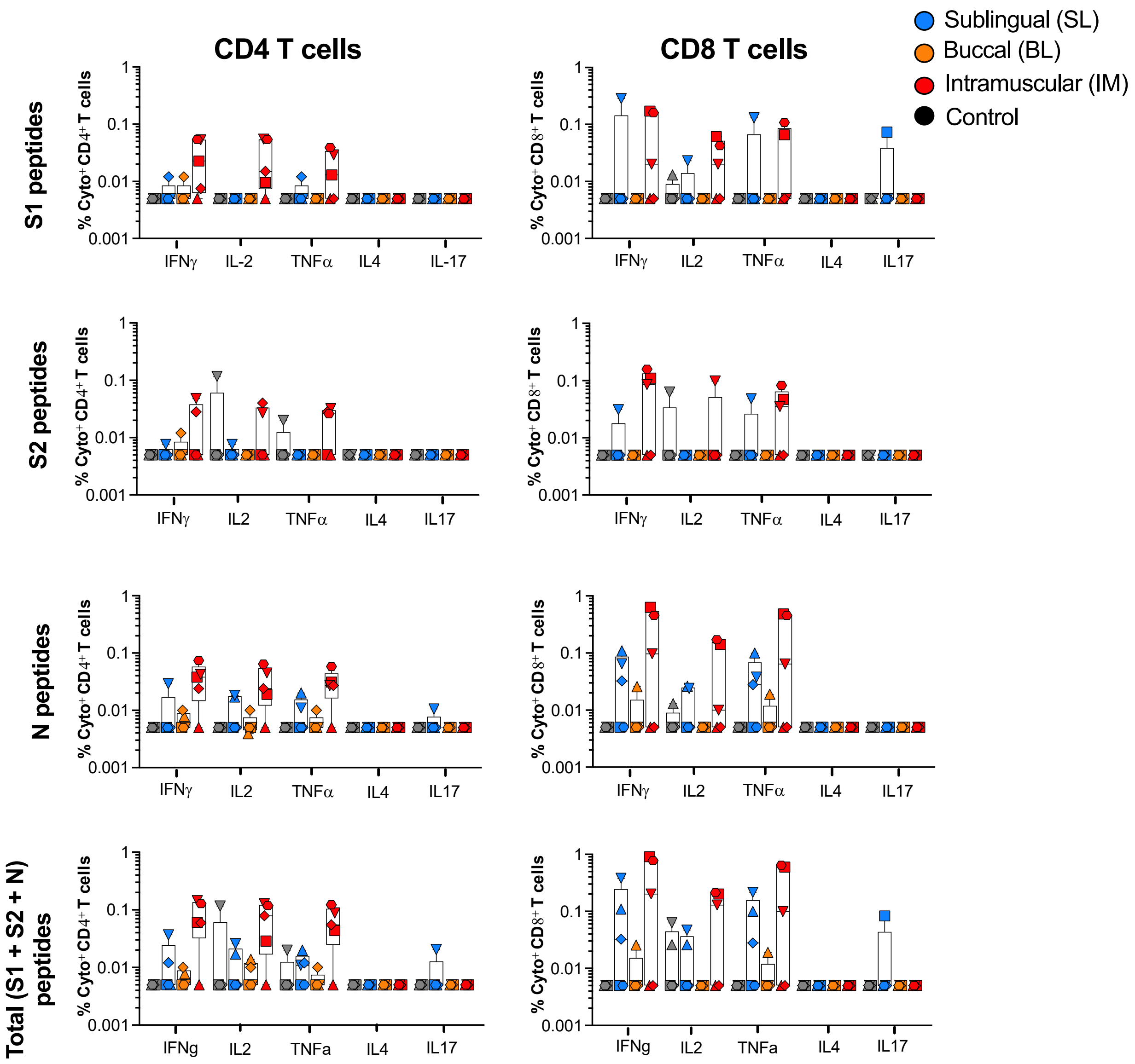

Figure S4. The MVA/SdFCS-N vaccination induced T cell responses in the blood of NHPs. Cytokine positive+ CD4+ (left) and CD8+ (right) T cells in blood after MVA/SdFCS-N (Week 5, 1-week after boost) vaccination, after re-stimulation with a peptide pool specific to the indicated protein. S1 peptides (S1 region of spike); S2 (S2 region of spike), N (nucleocapsid).

Figure S5

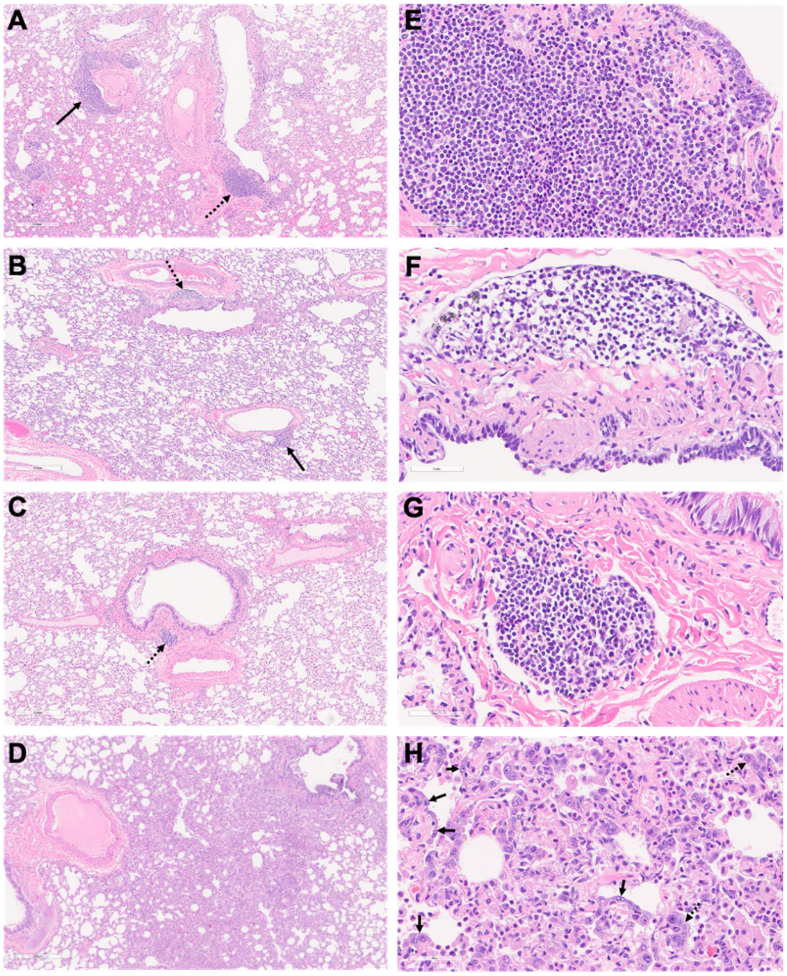

Figure S5. Post-challenge lung pathology in rhesus macaques. Representative hematoxylin and eosin-stained images of lung Pathology of rhesus macaques infected with SARS-CoV-2 delta (B.1.617.2). Left-side panel (Panels A-D) are low magnification (500 µm scale bar, 20x magnification) and right-side panel (Panels E-H) are high magnification (50 µm scale bar, 200x magnification). NHPs vaccinated with MVA/SdFCS-N via SL (A and E), BU (B and F), IM (C and G), or Control MVA/Empty (D and H), following SARS-CoV-2 challenge, and multiple sections were scanned for pathological features and then the representative image with 20x and 200x magnification is shown. Low magnification photomicrograph of perivascular (solid arrow) and peribronchiolar (dashed arrow) lymphoid aggregates in RTp18 (A), inflammatory aggregates in RDk18 (B), in RBv18 (C), a consolidated area in the lung parenchyma in RLq18 (D). High magnification photomicrograph of depicting a peribronchiolar inflammatory aggregate consisting of lymphocytes, histiocytes and lesser numbers of plasma cells in RTp18 (E), in RDk18 (B), in RBv18 (C), and depicting type II pneumocyte hyperplasia (solid arrows) and mild thickening of alveolar walls throughout the image in RLq18 (D). See also supplementary Table 1.

Figure S6

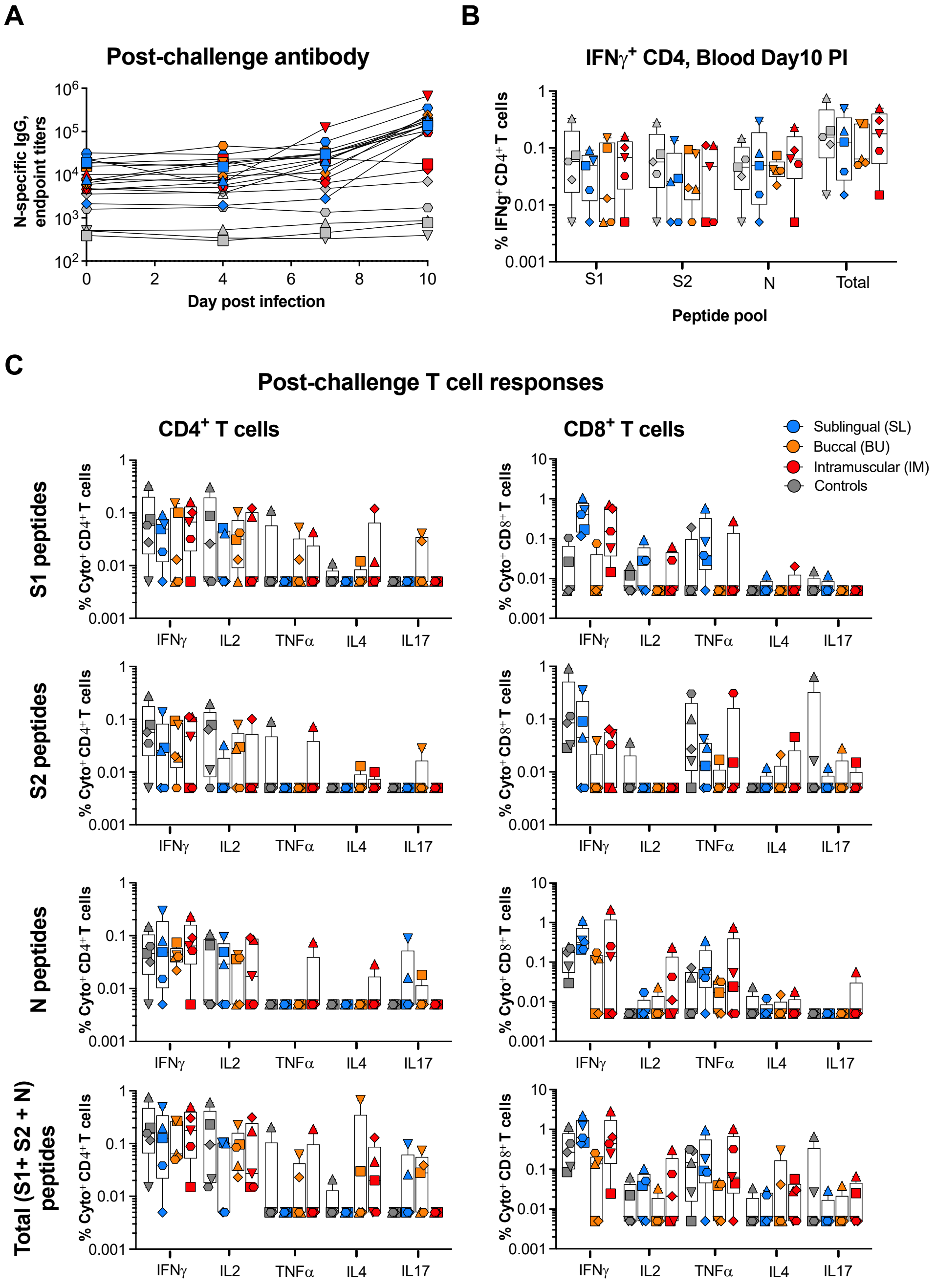

Figure S6. Nucleocapsid-specific antibody responses and T cell responses in the blood induced in NHPs following delta virus challenge. (A) N (WA-1/2020)-specific serum IgG responses following challenge. Data represent one independent experiment. Each sample was analyzed in duplicate and repeated two independent times. (B) S1, S2 and N-specific CD4+ T cells in blood at day 10 post-infection after re-stimulation with a peptide pool. (**C**) Cytokine positive+ CD4+ and CD8+ T cells in blood after delta virus challenge (10-days after challenge), after re-stimulation with a peptide pool specific to the indicated protein. S1 peptides (S1 region of spike); S2 (S2 region of spike), N (nucleocapsid).

Figure S7

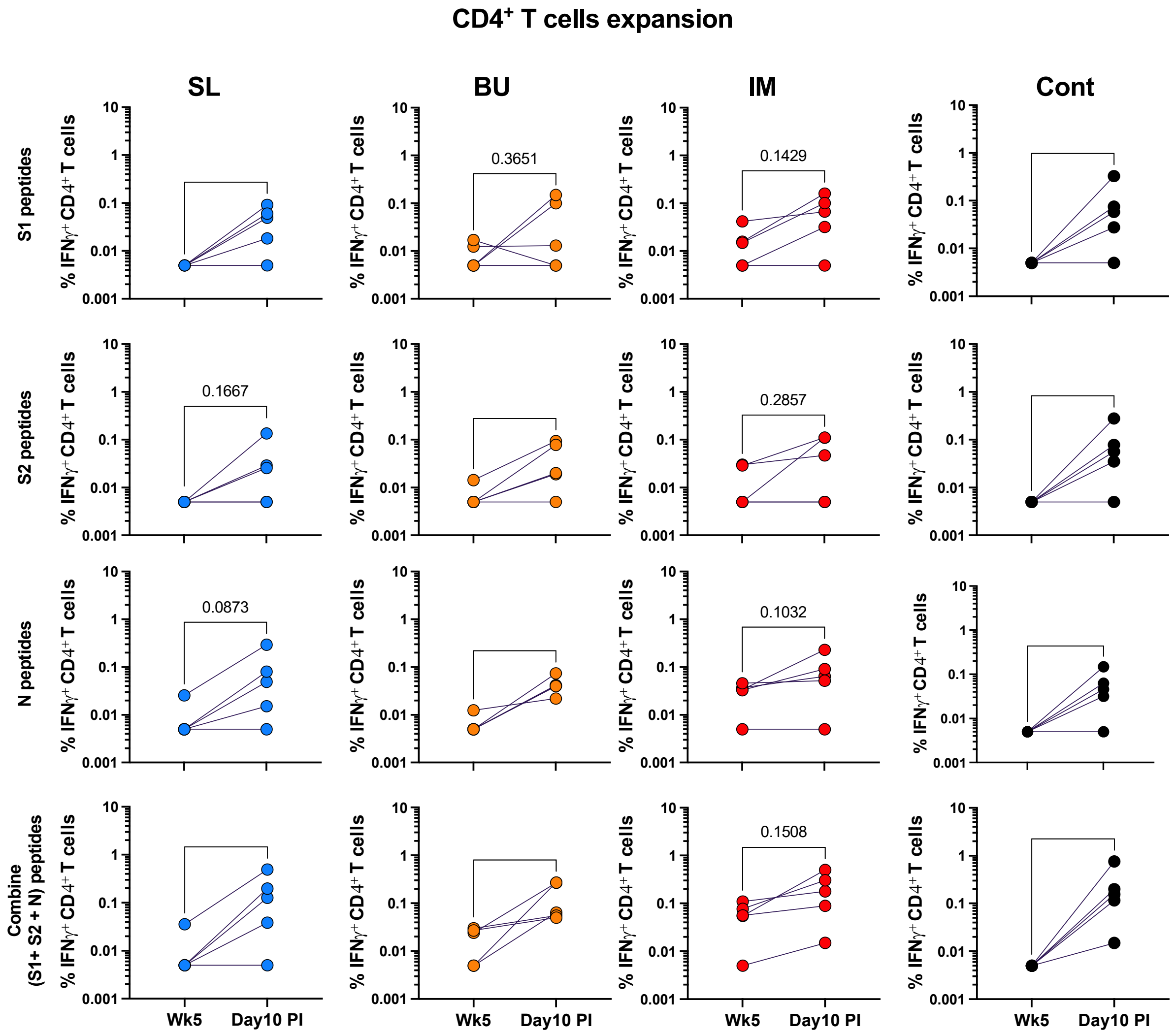

Figure S7. CD4+ T cell expansion following SARS-CoV-2 delta challenge, compared to post-vaccination (Week 5, 1-week after boost) responses in the blood of NHPs. IFNγ+ CD4+ T cells in blood before (Week 5, 1-week after boost) and after delta virus challenge (10-days after challenge), after re-stimulation with a peptide pool specific to the indicated protein. S1 peptides (S1 region of spike); S2 (S2 region of spike), N (nucleocapsid).

Figure S8

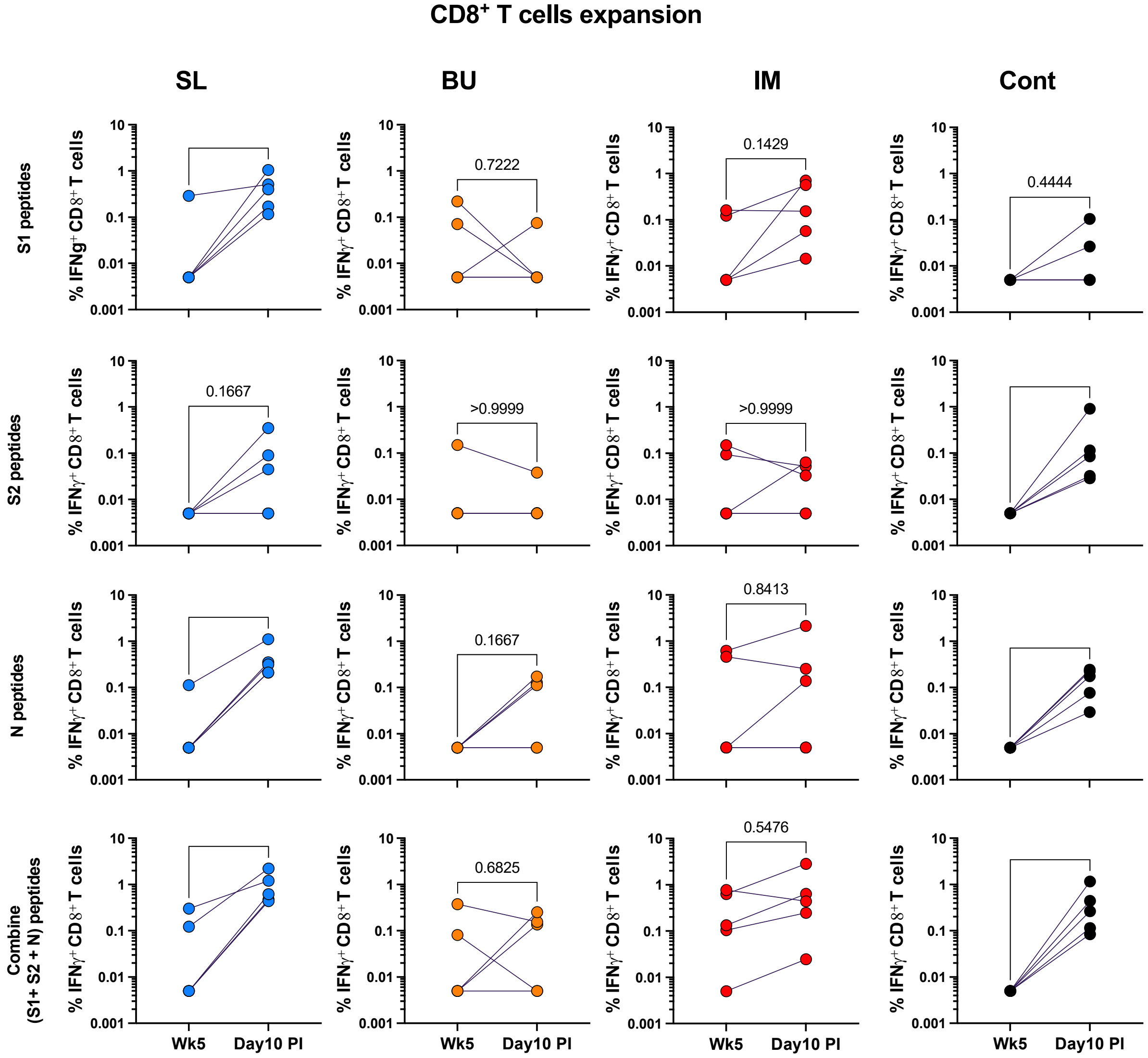

Figure S8. CD8+ T cell expansion following SARS-CoV-2 delta challenge, compared to post-vaccination (Week 5, 1-week after boost) responses in the blood of NHPs. IFNγ+ CD8+ T cells in blood before (Week 5, 1-week after boost) and after delta virus challenge (10-days after challenge), after re-stimulation with a peptide pool specific to the indicated protein. S1 peptides (S1 region of spike); S2 (S2 region of spike), N (nucleocapsid).

Figure S9

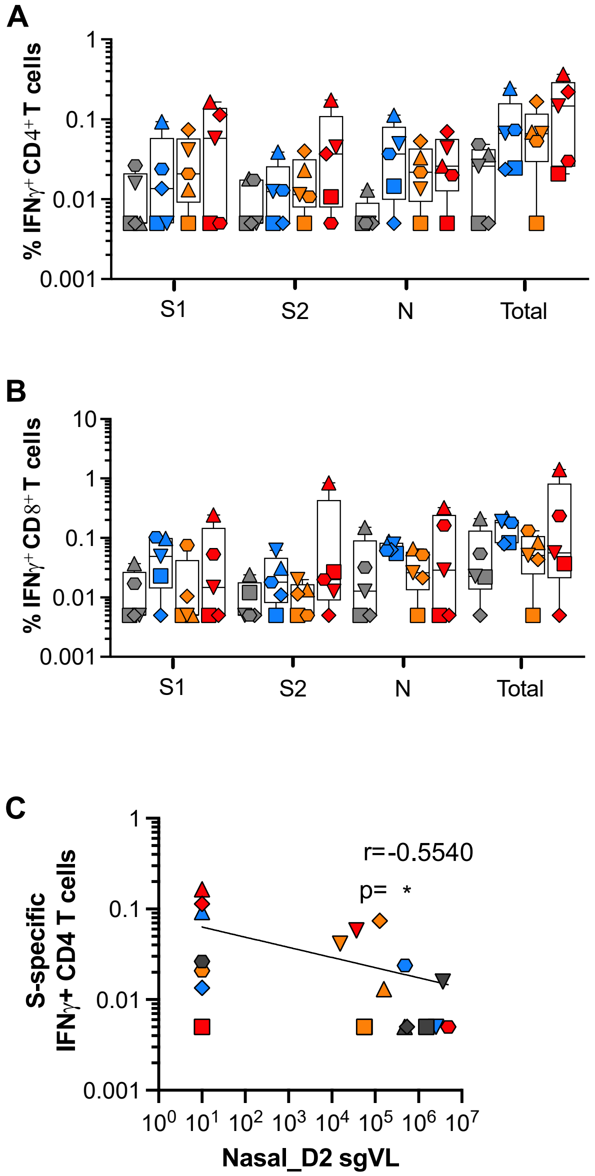

Figure S9. Spike- and N-specific CD4 and CD8 T cell responses in the hilar lymph node following SARS-CoV-2 delta challenge in macaques. S1, S2 and N-specific **(A)** CD4+ and **(B)** CD8+ T cells in lung draining lymph node (hLN) at day 10 post-infection after stimulation with respective peptide pool. **C**) correlation between S1-specific IFNγ+ CD4 T cells in the hilar LN and day 2 viral loads in the nose. S1, S1 region of spike amino acid residues 1-685; S2, S2 region of spike amino acid residues 686-1273; N, nucleocapsid; Total, total response (S1 + S2 + N).

Table S1

NHP Group	Necropsy ID	Animal ID	Type 2 pneumocyte hyperplasia	Alveolar septal thickening	Fibrosis	Perivascular cuffing	Peribronchiolar hyperplasia	Total score
**MVA/SdFCS-N, Sublingual (SL)**	YN21-313	RTz18	0	0	0	3	9	12
	YN21-325	RTp18	0	0	0	3	9	12
	YN21-338	RMi18	0	0	0	5	5	10
	YN21-344	RFy18	0	0	0	2	6	8
	YN21-312	RRk18	0	0	0	0	7	7
**MVA/SdFCS-N, Buccal (BU)**	YN21-314	RLy18	0	0	0	3	6	9
	YN21-326	RRj18	0	0	0	1	6	7
	YN21-339	RBl18	0	0	0	1	5	6
	YN21-345	RMw18	0	0	0	6	3	9
	YN21-324	RDk18	0	0	0	1	4	5
**MVA/SdFCS-N, Intramuscular (IM)**	YN21-310	RBv18	0	0	0	0	5	5
	YN21-322	RBu18	0	0	0	0	5	5
	YN21-335	RHk18	0	0	0	1	6	7
	YN21-341	RMy18	0	0	0	0	6	6
	YN21-337	RLu18	0	0	0	6	11	17
**Control (MVA/Empty), Intramuscular (IM)**	YN21-311	RCs18	0	0	0	0	2	2
	YN21-323	RLm18	3	2	0	4	5	14
	YN21-336	RLq18	12	12	0	6	6	36
	YN21-342	RKj18	0	0	0	0	6	6
	YN21-343	RUw17	0	0	0	4	6	10

Table S1. Lung pathology scores of individual animals at necropsy (day 10 post infection).

References and Notes

1. N. K. Routhu, N. Cheedarla, S. Gangadhara, V. S. Bollimpelli, A. K. Boddapati, A. Shiferaw, S. A. Rahman, A. Sahoo, V. V. Edara, L. Lai, K. Floyd, S. Wang, S. Fischinger, C. Atyeo, S. A. Shin, S. Gumber, S. Kirejczyk, J. Cohen, S. M. Jean, J. S. Wood, F. Connor-Stroud, R. L. Stammen, A. A. Upadhyay, K. Pellegrini, D. Montefiori, P. Y. Shi, V. D. Menachery, G. Alter, T. H. Vanderford, S. E. Bosinger, M. S. Suthar, R. R. Amara, A modified vaccinia Ankara vector-based vaccine protects macaques from SARS-CoV-2 infection, immune pathology, and dysfunction in the lungs. *Immunity* **54**, 542-556 e549 (2021).
2. N. K. Routhu, N. Cheedarla, V. S. Bollimpelli, S. Gangadhara, V. V. Edara, L. Lai, A. Sahoo, A. Shiferaw, T. M. Styles, K. Floyd, S. Fischinger, C. Atyeo, S. A. Shin, S. Gumber, S. Kirejczyk, K. H. Dinnon, 3rd, P. Y. Shi, V. D. Menachery, M. Tomai, C. B. Fox, G. Alter, T. H. Vanderford, L. Gralinski, M. S. Suthar, R. R. Amara, SARS-CoV-2 RBD trimer protein adjuvanted with Alum-3M-052 protects from SARS-CoV-2 infection and immune pathology in the lung. *Nat Commun* **12**, 3587 (2021).
3. C. Garrido, A. D. Curtis, 2nd, M. Dennis, S. H. Pathak, H. Gao, D. Montefiori, M. Tomai, C. B. Fox, P. A. Kozlowski, T. Scobey, J. E. Munt, M. L. Mallory, P. T. Saha, M. G. Hudgens, L. C. Lindesmith, R. S. Baric, O. M. Abiona, B. Graham, K. S. Corbett, D. Edwards, A. Carfi, G. Fouda, K. K. A. Van Rompay, K. De Paris, S. R. Permar, SARS-CoV-2 vaccines elicit durable immune responses in infant rhesus macaques. *Sci Immunol* **6**, (2021).
4. P. A. Kozlowski, R. M. Lynch, R. R. Patterson, S. Cu-Uvin, T. P. Flanigan, M. R. Neutra, Modified wick method using Weck-Cel sponges for collection of human rectal secretions and analysis of mucosal HIV antibody. *Journal of acquired immune deficiency syndromes (1999)* **24**, 297-309 (2000).
5. X. Xie, A. Muruato, K. G. Lokugamage, K. Narayanan, X. Zhang, J. Zou, J. Liu, C. Schindewolf, N. E. Bopp, P. V. Aguilar, K. S. Plante, S. C. Weaver, S. Makino, J. W. LeDuc, V. D. Menachery, P. Y. Shi, An Infectious cDNA Clone of SARS-CoV-2. *Cell Host Microbe* **27**, 841-848 e843 (2020).
6. L. C. Katzelnick, A. Coello Escoto, B. D. McElvany, C. Chavez, H. Salje, W. Luo, I. Rodriguez-Barraquer, R. Jarman, A. P. Durbin, S. A. Diehl, D. J. Smith, S. S. Whitehead, D. A. T. Cummings, Viridot: An automated virus plaque (immunofocus) counter for the measurement of serological neutralizing responses with application to dengue virus. *PLoS Negl Trop Dis* **12**, e0006862 (2018).
7. E. P. Brown, K. G. Dowell, A. W. Boesch, E. Normandin, A. E. Mahan, T. Chu, D. H. Barouch, C. Bailey-Kellogg, G. Alter, M. E. Ackerman, Multiplexed Fc array for evaluation of antigen-specific antibody effector profiles. *J Immunol Methods* **443**, 33-44 (2017).
8. M. E. Ackerman, B. Moldt, R. T. Wyatt, A. S. Dugast, E. McAndrew, S. Tsoukas, S. Jost, C. T. Berger, G. Sciaranghella, Q. Liu, D. J. Irvine, D. R. Burton, G. Alter, A robust, high-throughput assay to determine the phagocytic activity of clinical antibody samples. *J Immunol Methods* **366**, 8-19 (2011).
9. S. Fischinger, J. K. Fallon, A. R. Michell, T. Broge, T. J. Suscovich, H. Streeck, G. Alter, A high-throughput, bead-based, antigen-specific assay to assess the ability of antibodies to induce complement activation. *J Immunol Methods* **473**, 112630 (2019).
10. C. B. Karsten, N. Mehta, S. A. Shin, T. J. Diefenbach, M. D. Slein, W. Karpinski, E. B. Irvine, T. Broge, T. J. Suscovich, G. Alter, A versatile high-throughput assay to characterize antibody-mediated neutrophil phagocytosis. *J Immunol Methods* **471**, 46-56 (2019).
11. M. S. Parsons, W. S. Lee, A. B. Kristensen, T. Amarasena, G. Khoury, A. K. Wheatley, A. Reynaldi, B. D. Wines, P. M. Hogarth, M. P. Davenport, S. J. Kent, Fc-dependent functions

are redundant to efficacy of anti-HIV antibody PGT121 in macaques. *J Clin Invest* **129**, 182-191 (2019).
12. R. R. Amara, F. Villinger, J. D. Altman, S. L. Lydy, S. P. O'Neil, S. I. Staprans, D. C. Montefiori, Y. Xu, J. G. Herndon, L. S. Wyatt, M. A. Candido, N. L. Kozyr, P. L. Earl, J. M. Smith, H. L. Ma, B. D. Grimm, M. L. Hulsey, J. Miller, H. M. McClure, J. M. McNicholl, B. Moss, H. L. Robinson, Control of a mucosal challenge and prevention of AIDS by a multiprotein DNA/MVA vaccine. *Science* **292**, 69-74 (2001).
13. R. Wolfel, V. M. Corman, W. Guggemos, M. Seilmaier, S. Zange, M. A. Muller, D. Niemeyer, T. C. Jones, P. Vollmar, C. Rothe, M. Hoelscher, T. Bleicker, S. Brunink, J. Schneider, R. Ehmann, K. Zwirglmaier, C. Drosten, C. Wendtner, Virological assessment of hospitalized patients with COVID-2019. *Nature* **581**, 465-469 (2020).
14. J. J. Waggoner, V. Stittleburg, R. Pond, Y. Saklawi, M. K. Sahoo, A. Babiker, L. Hussaini, C. S. Kraft, B. A. Pinsky, E. J. Anderson, N. Rouphael, Triplex Real-Time RT-PCR for Severe Acute Respiratory Syndrome Coronavirus 2. *Emerg Infect Dis* **26**, 1633-1635 (2020).
